# Supplementary material for: Autophagy induction promoted by m6A reader YTHDF3 through translation upregulation of FOXO3 mRNA
Source: Nat Commun. 2022 Oct 4;13:5845. doi: 10.1038/s41467-022-32963-0 (PMC9532426; doi:10.1038/s41467-022-32963-0)
Supplement: Supplementary file 1 — Supplementary Information [file 41467_2022_32963_MOESM1_ESM.pdf]

Supplementary information

Autophagy induction promoted by m^{6}A reader YTHDF3 through translation upregulation of FOXO3 mRNA

W. Hao, M. Dian, Y. Zhou, Q. Zhong, W. Pang, Z. Li, Y. Zhao, J. Ma, X. Lin, R. Luo, Y. Li, J. Jia, H. Shen, S. Huang, G. Dai, J. Wang, Y. Sun, D. Xiao

*Corresponding authors. Email: xiaodong@smu.edu.cn (D.X.); sunyan@gdph.org.cn (Y.S.); wjh1987@smu.edu.cn (J.W.)

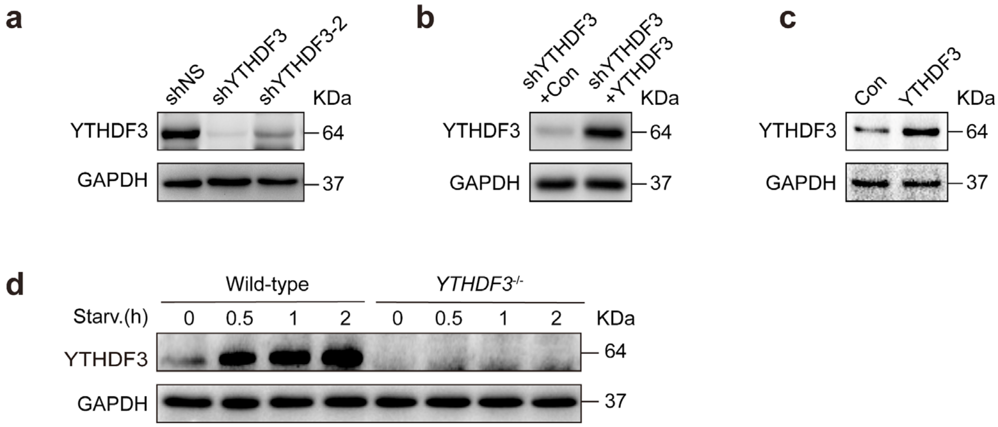

Supplementary Fig.1. Immunoblot analyses of YTHDF3 expression in MEF stable cell lines, as well as wild-type and *YTHDF3*$^{-/-}$ MEFs. **a** Immunoblot analyses of YTHDF3 in MEFs infected with either nonspecific shRNA (shNS) or two independent shRNAs targeting YTHDF3 (shYTHDF3 and shYTHDF3-2). **b** Immunoblot analyses of YTHDF3 in shYTHDF3 MEFs ectopically expressing either YTHDF3 (shYTHDF3+YTHDF3) or its control vector (shYTHDF3+Con). **c** Immunoblot analyses of YTHDF3 overexpression and control MEFs. **d** Immunoblot analyses of YTHDF3 in wild-type and *YTHDF3*$^{-/-}$ MEFs following nutrient starvation for the indicated time periods. GAPDH are shown as loading controls. Source data are provided as a Source Data file.

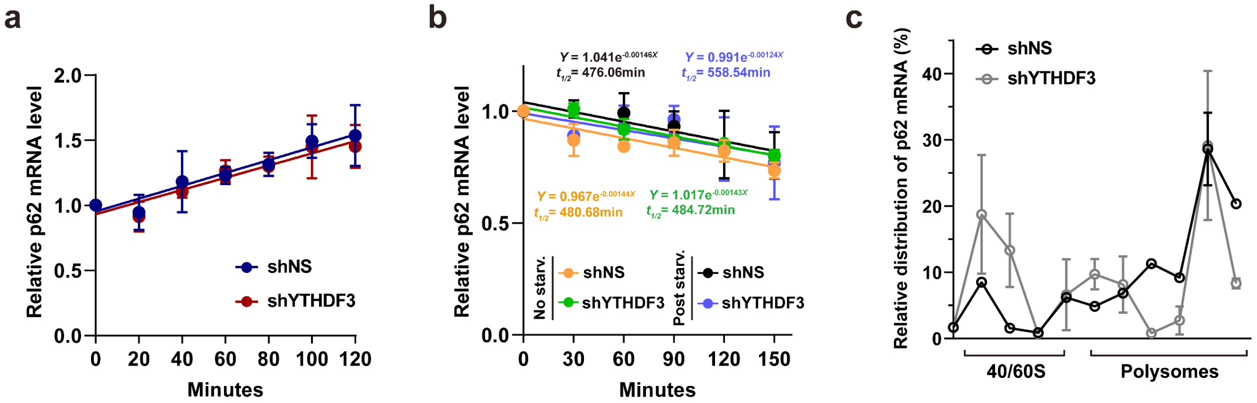

Supplementary Fig.2. p62 mRNA expression in shNS and shYTHDF3 MEFs. a qRT-PCR analyses of p62 in shNS and shYTHDF3 MEFs following nutrient starvation for the indicated time periods. **b** shNS and shYTHDF3 MEFs were treated with Act.D (5μg/mL) for the indicated time periods, with or without nutrient starvation, respectively. The expression of p62 was examined with qRT-PCR. **c** Relative levels of p62 mRNAs in each ribosome fraction were quantified and plotted as a percentage relative to the total input. Data from three independent experiments are presented as mean values ± SEM. Source data are provided as a Source Data file.

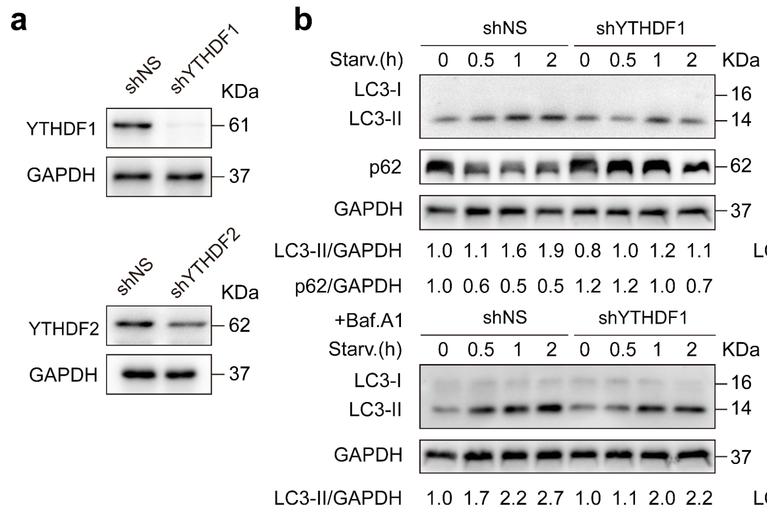

Supplementary Fig.3. Immunoblot analyses of the impact of YTHDF1 or YTHDF2 depletion on autophagy flux. a Immunoblot analyses of YTHDF1 in shNS and shYTHDF1 MEFs, and YTHDF2 in shNS and shYTHDF2 MEFs. **b, c** Immunoblot analyses of LC3-II and p62 in shNS and shYTHDF1 MEFs (b), as well as shNS and shYTHDF2 MEFs (c), following nutrient starvation for the indicated time periods, with and without Baf.A1 treatment (20nM). GAPDH are shown as loading controls. Source data are provided as a Source Data file.

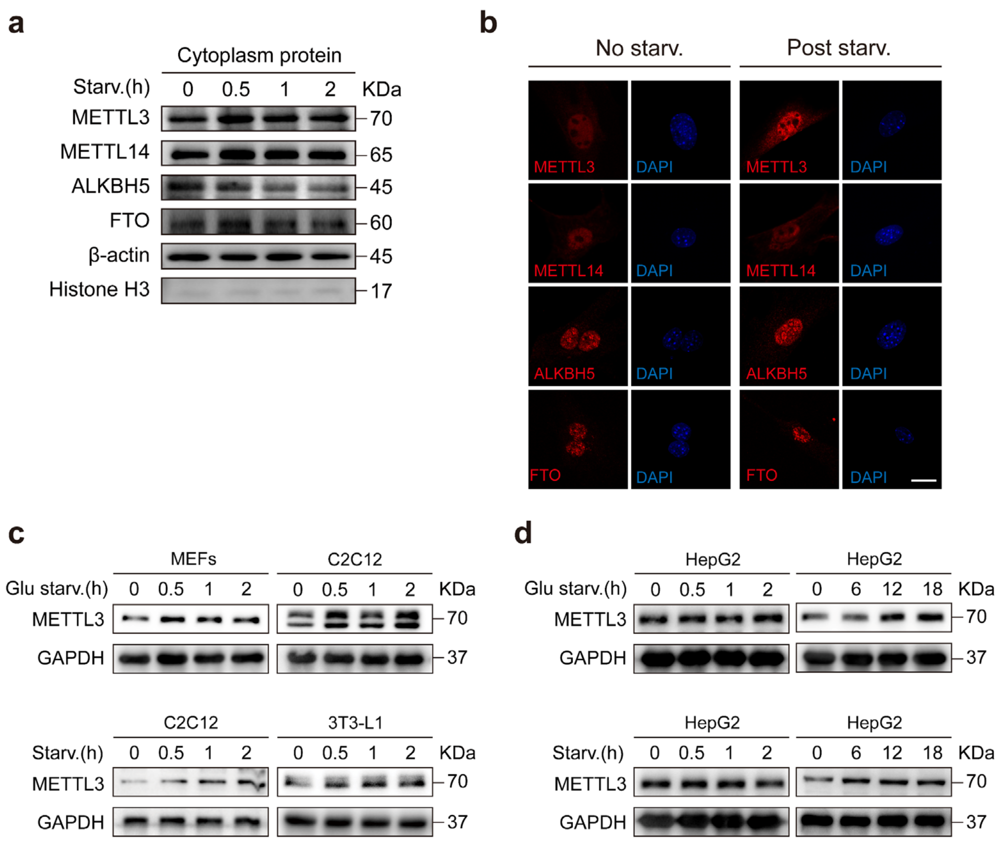

Supplementary Fig.4. Detection of the m^{6}A writer and eraser proteins in MEFs during nutrient deficiency. a Cytoplasm fractions from MEFs following nutrient starvation for the indicated time periods were subjected to immunoblotting. **b** MEFs following nutrient starvation for the indicated time periods were subjected to fluorescence microscopy. Endogenous METTL3, METTL14, ALKBH5, and FTO were stained. Nuclei were stained with DAPI. Scale bar, 20μm. **c, d** The indicated cells were starved in either the glucose-deprived medium or HBSS for the indicated time periods, followed by immunoblot analyses of METTL3. Source data are provided as a Source Data file.

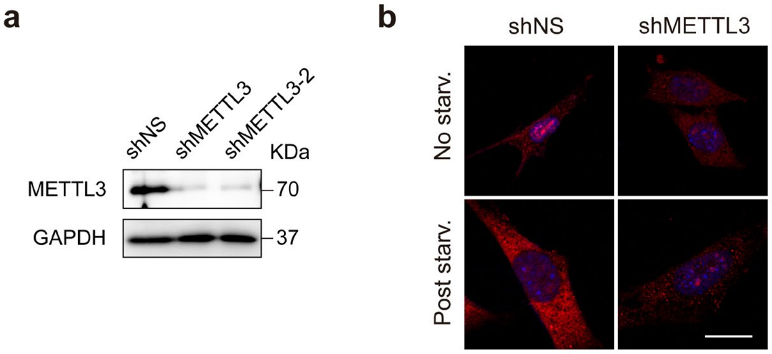

Supplementary Fig.5. Representative confocal images of m^{6}A fluorescence localization in shNS and shMETTL3 MEFs. a Immunoblot analyses of METTL3 in MEFs infected with shNS or two independent METTL3 shRNAs (shMETTL3 and shMETTL3-2). **b** Representative confocal images of m^{6}A fluorescence localization were obtained in shNS and shMETTL3 MEFs, with or without nutrient deprivation. Nuclei were stained with DAPI. Scale bar, 20μm. Source data are provided as a Source Data file.

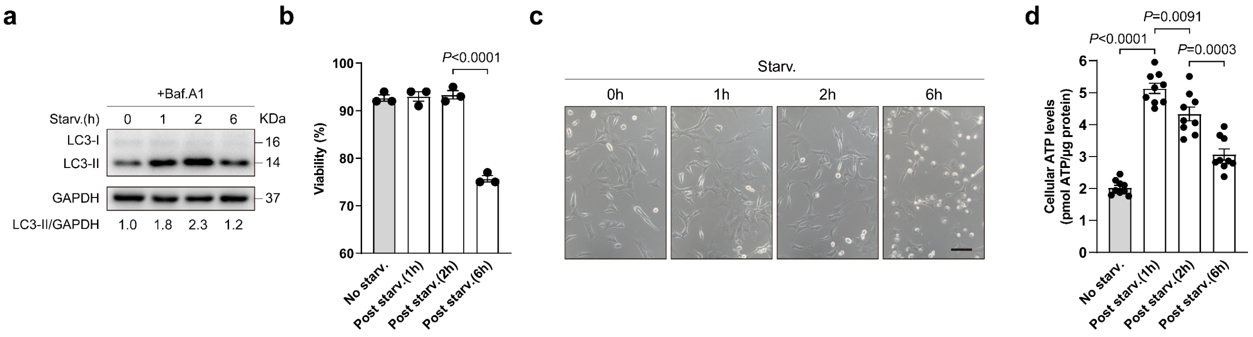

Supplementary Fig.6. Transient activation of METTL3 favors cell survival and metabolism. **a** Immunoblot analysis of LC3-II in MEFs following nutrient starvation for the indicated time periods with Baf.A1 treatment(20nM). GAPDH was used as a loading control. **b** Percentage of surviving cells after nutrient starvation for the indicated time periods. **c** Representative phase-contrast images of MEFs following nutrient starvation for the indicated time periods. Scale bar, 100μm. **d** Measurement of cellular ATP levels in MEFs following nutrient starvation for the indicated time periods. For **b**, **d**, data from three independent experiments are presented as mean values ± SEM. Two-tailed unpaired *t*-tests were used to estimate significance. *P*-values are indicated in the figure. Source data are provided as a Source Data file.

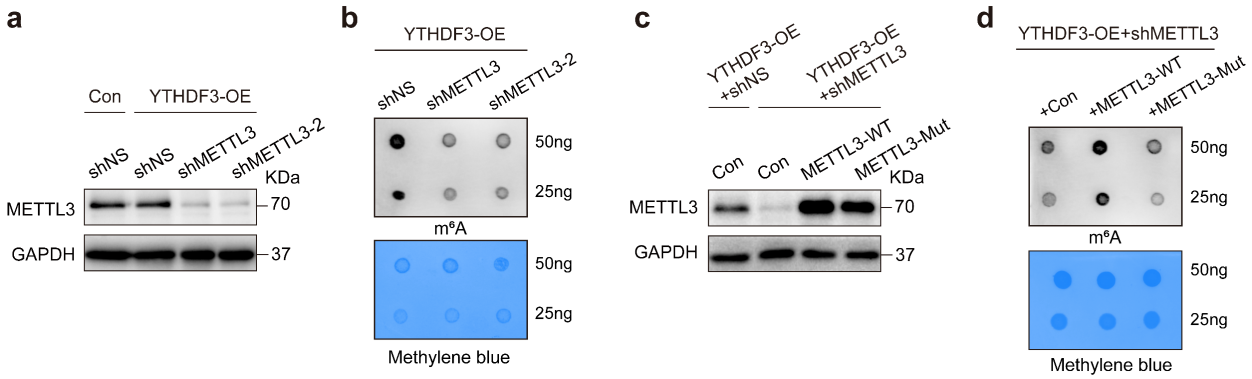

Supplementary Fig.7. Immunoblot analyses and m^{6}A dot blot analyses of YTHDF3-OE MEFs infected with shNS or two independent METTL3 shRNAs (shMETTL3 and shMETTL3-2). a Immunoblot analyses of YTHDF3-OE MEFs infected with shNS or two independent METTL3 shRNAs (shMETTL3 and shMETTL3-2). **b** Poly(A)+ RNA from samples in **a** were subjected to dot blot analysis with an antibody recognizing m^{6}A. Loading control was methylene blue. **c** Immunoblot analyses of METTL3-silenced YTHDF3-OE MEFs transfected with lentiviral vectors (Con), wild-type METTL3 (METTL3-WT), or a catalytic mutant of METTL3 (METTL3-Mut). **d** Poly(A)+ RNA from samples in **c** were subjected to dot blot analysis with an antibody recognizing m^{6}A. Loading control was methylene blue. Source data are provided as a Source Data file.

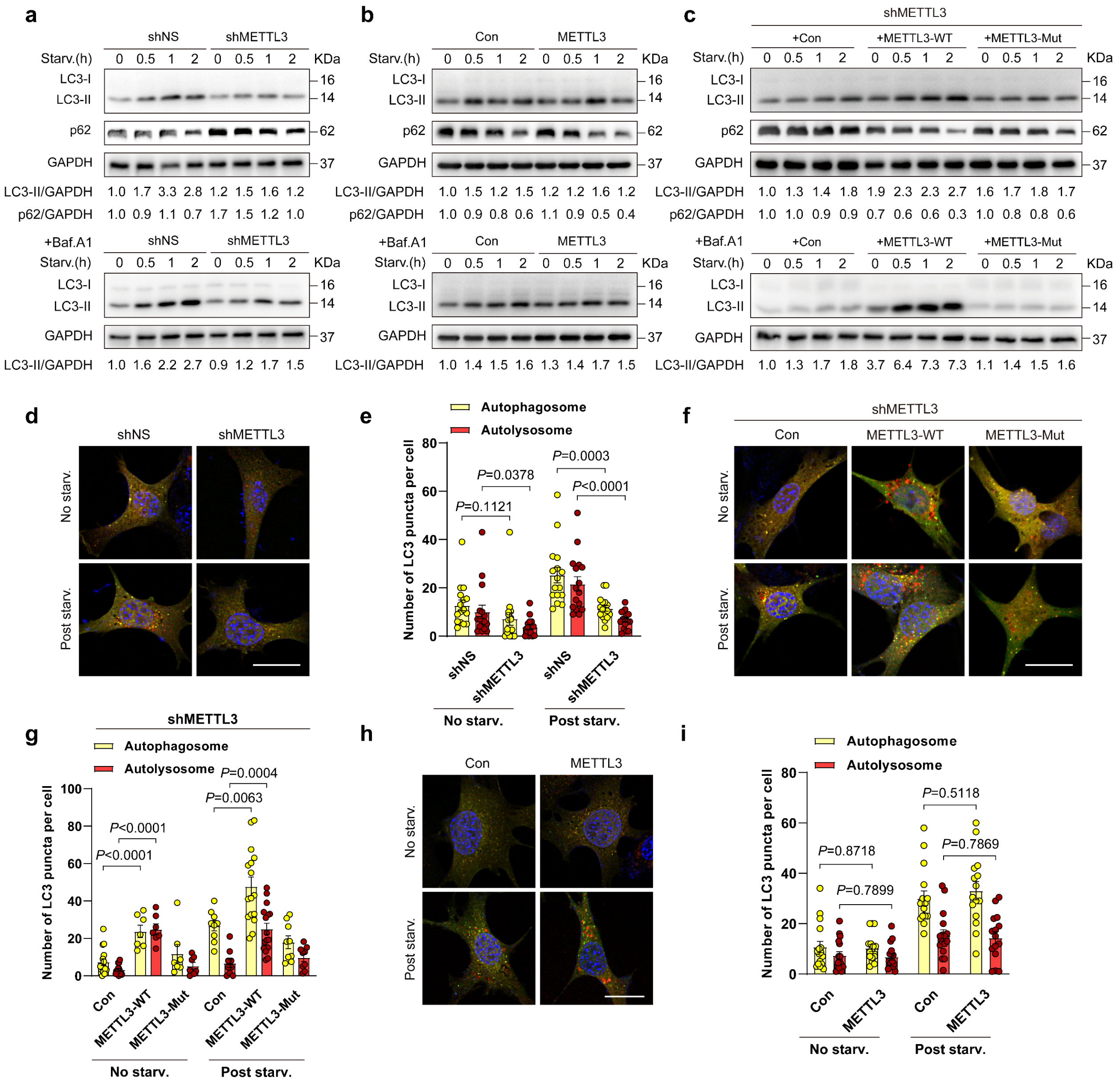

Supplementary Fig.8. Detection of the METTL3's impact on autophagy flux in MEFs without YTHDF3 overexpression. **a** Immunoblot analyses of LC3-II and p62 in shNS and shMETTL3 MEFs following nutrient starvation for the indicated time periods, with or without Baf.A1 treatment (20nM). GAPDH was used as a loading control. **b** Immunoblot analyses of LC3-II and p62 in MEFs ectopically expressing either METTL3 or its control vector (Con). GAPDH is shown as a loading control. **c** Immunoblot analyses of METTL3-silenced MEFs transfected with lentiviral vectors (Con), wild type METTL3 (METTL3-WT), or a catalytic mutant of METTL3 (METTL3-Mut) following nutrient starvation for the indicated time periods, with or without Baf.A1 treatment (20nM). GAPDH is used as a loading control. **d, e** Measurement of autophagy flux and quantification of autophagosomes (yellow) and autolysosomes (red) by a tandem mCherry-GFP-LC3 reporter assay in shNS and shMETTL3 MEFs. Scale bar, 20μm. **f, g** Measurement of autophagy flux and quantification of

autophagosomes (yellow) and autolysosomes (red) by a tandem mCherry-GFP-LC3 reporter assay in METTL3-silenced MEFs transfected with METTL3-WT, METTL3-Mut, or Con, with or without nutrient deficiency. **h, i** Measurement of autophagy flux and quantification of autophagosomes (yellow) and autolysosomes (red) by a tandem mCherry-GFP-LC3 reporter assay in MEFs ectopically expressing either METTL3 or its control vector (Con) with or without nutrient deficiency. Scale bar, 20μm. For **e**, **g**, **i**, mean numbers of puncta per cell from each randomly selected field over three independent experiments were plotted (dots). Bars represent mean values ± SEM. Two-tailed unpaired multiple *t*-tests with two-stage step-up correction (Benjamini, Krieger, and Yekutieli) were used to estimate significance. *P*-values are indicated in the figure. Source data are provided as a Source Data file.

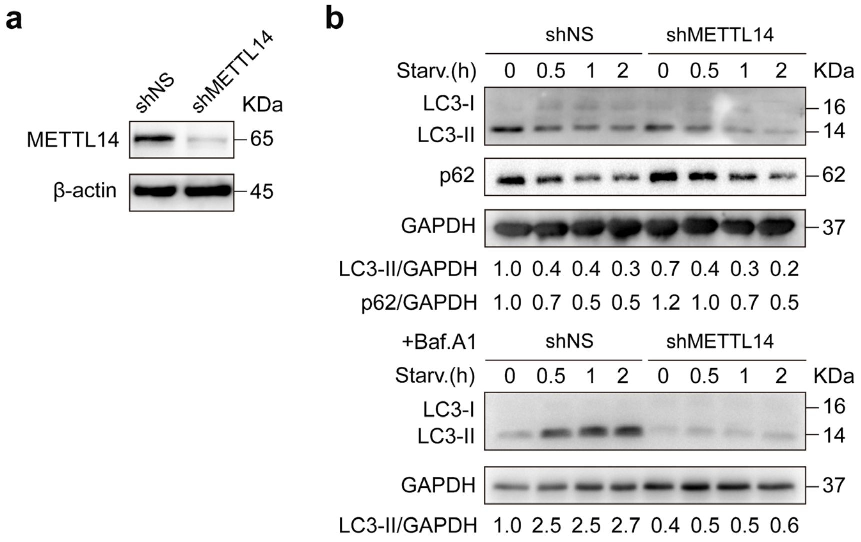

Supplementary Fig.9. Immunoblot analysis of the impact of METTL14 depletion on autophagy flux. a Immunoblot analyses of METTL14 in shNS and shMETTL14 MEFs. **b** Immunoblot analyses of LC3-II and p62 in shNS and shMETTL14 MEFs following nutrient starvation for the indicated time periods, with and without Baf.A1 treatment (20nM). GAPDH is used as a loading control. Source data are provided as a Source Data file.

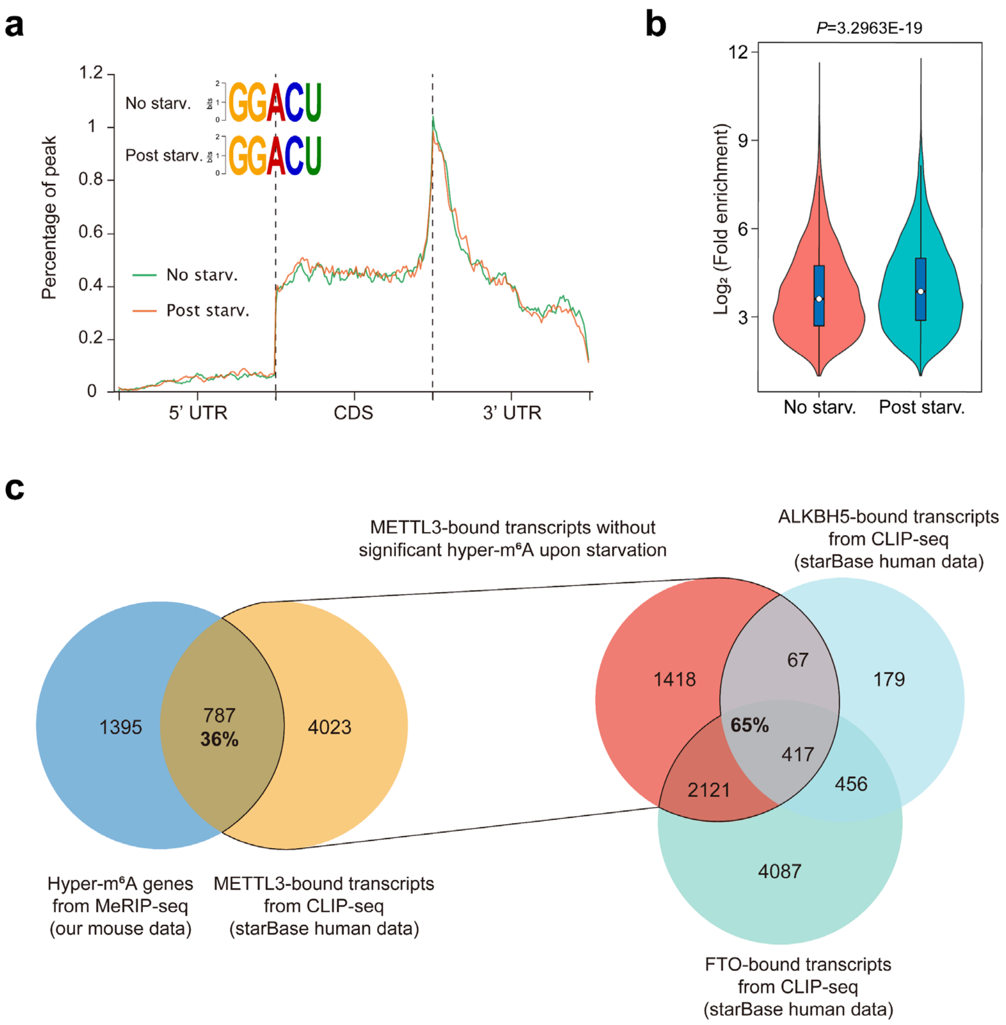

Supplementary Fig.10. MeRIP-seq data analyses. a Metagene plots depict the distribution of m^{6}A peaks in MEFs, with or without nutrient starvation. The consensus motifs within m^{6}A peaks were generated by DREME database. **b** Violin box plots of log2 fold enrichment of all the m^{6}A peaks in each group (n=2 biological replicates). Violin plot range denotes the minima and maxima, boxes represent 25th and 75th percentile, centre depicts the median, and whiskers indicate 1.5×interquartile range. *P*=3.2963E-19 by two-tailed Wilcoxon signed-rank tests. **c** Venn diagrams show overlap between hyper-m^{6}A genes from our MeRIP-seq data and METTL3-bound genes from starBase human CLIP-seq data; and overlap between the resultant METTL3-bound genes which were not hyper-m^{6}A-methylated upon nutrient starvation and FTO- or ALKBH5-bound genes from starBase human CLIP-seq data.

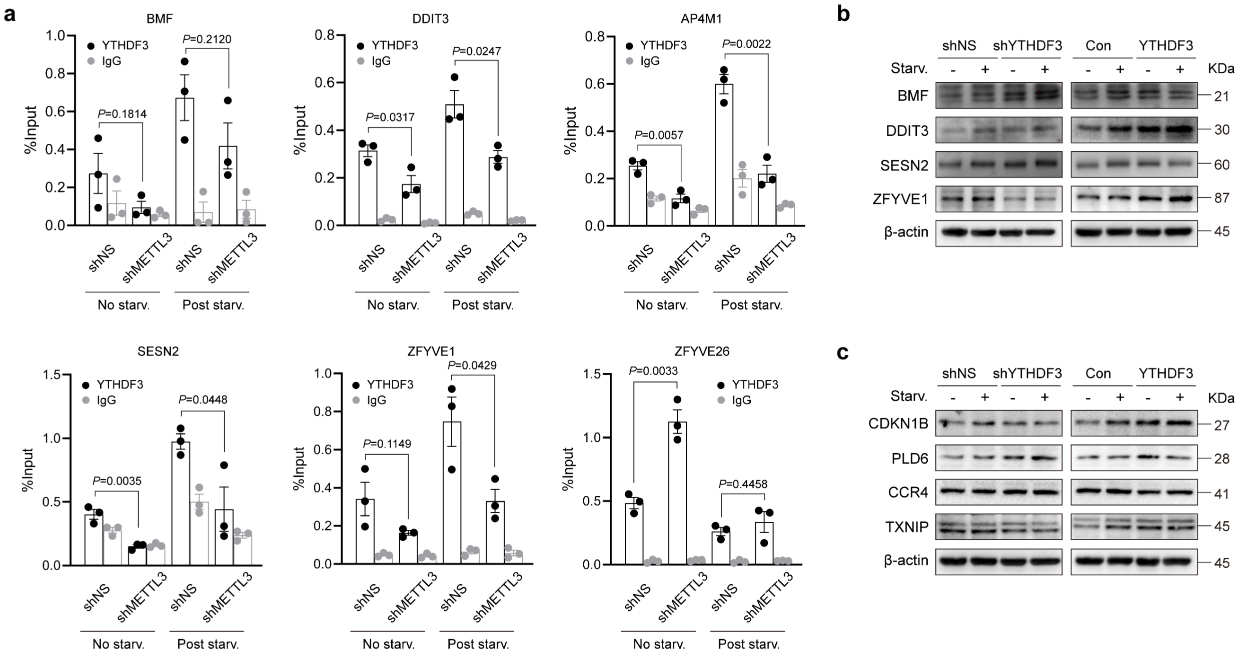

Supplementary Fig.11. Identification of FOXO3 as the candidate of YTHDF3. a YTHDF3-RIP followed by qRT-PCR detected the interaction between YTHDF3 and the indicated mRNAs in shNS and shMETTL3 MEFs upon nutrient deficiency (n=3 biological replicates). Data are presented as mean values ± SEM. Two-tailed unpaired *t*-tests were used to estimate significance. *P*-values are indicated in the figure. **b, c** Immunoblot analyses of indicated proteins in shNS, shYTHDF3, control, and YTHDF3-OE MEFs, with or without nutrient starvation, respectively. Source data are provided as a Source Data file.

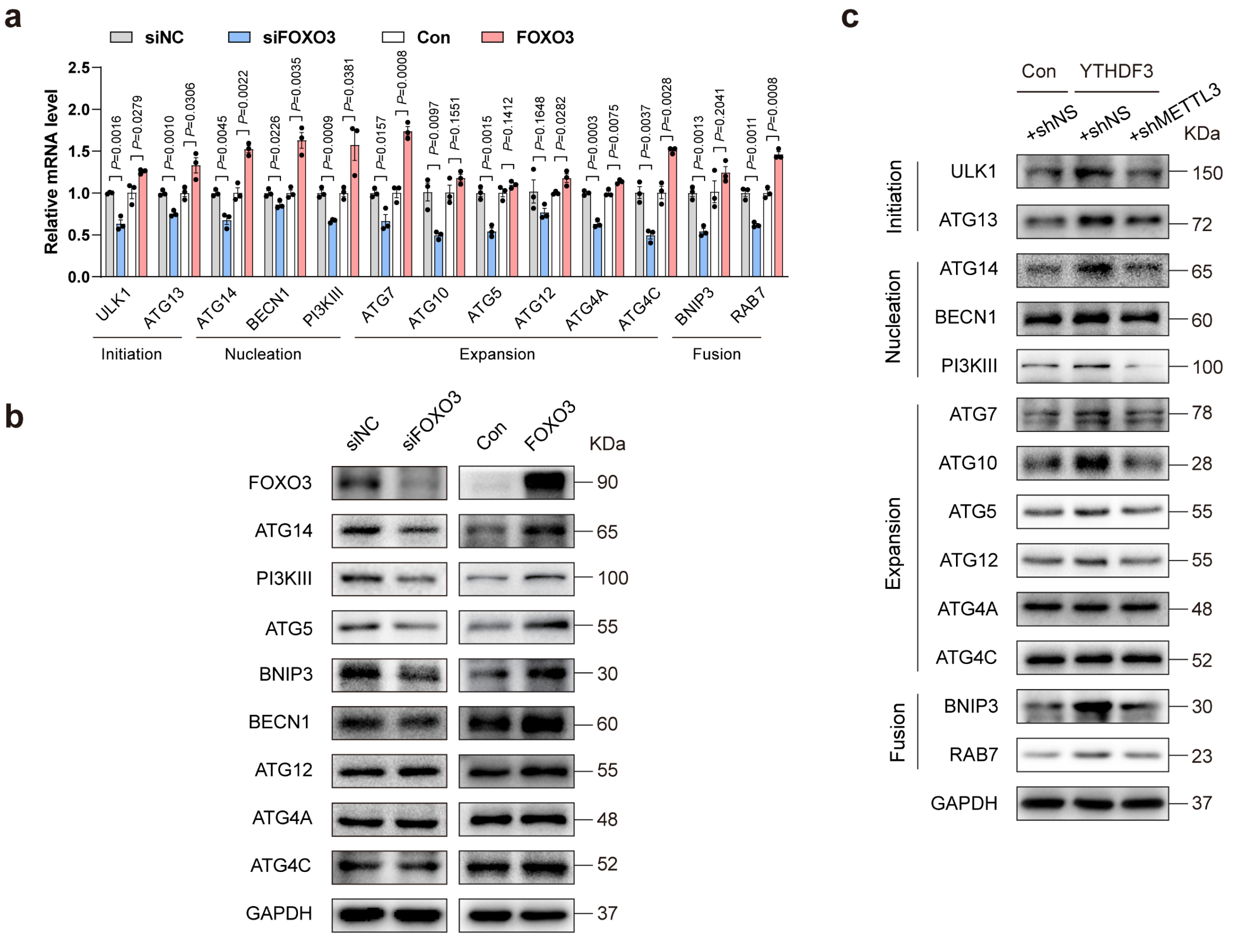

Supplementary Fig.12. YTHDF3-promoted FOXO3 targeted autophagic genes are dependent on METTL3. a qRT-PCR analysis of mRNA levels of FOXO3 target autophagy-related genes in siNC, siFOXO3, control, and FOXO3-OE MEFs (n=3 biological replicates). Bars represent mean ± SEM. Two-tailed unpaired multiple *t*-tests with two-stage step-up correction (Benjamini, Krieger, and Yekutieli) were used to estimate significance. *P*-values are indicated in the figure. **b** Immunoblot analyses of protein levels of the indicated FOXO3 targeted autophagic genes in siNC, siFOXO3, control, and FOXO3-OE MEFs. **c** Immunoblot analyses of protein levels of FOXO3 targeted autophagic genes in METTL3-silenced YTHDF3-OE MEFs. Source data are provided as a Source Data file.

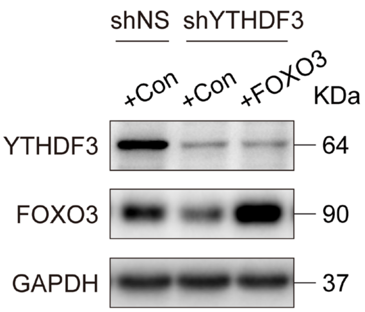

Supplementary Fig.13. Immunoblot analyses of FOXO3 rescued MEFs (shYTHDF3+FOXO3) and control MEFs (shYTHDF3+Con). Immunoblot analyses of YTHDF3 and FOXO3 in FOXO3 rescued MEFs (shYTHDF3+FOXO3) and control MEFs (shYTHDF3+Con). GAPDH is used as a loading control. Source data are provided as a Source Data file.

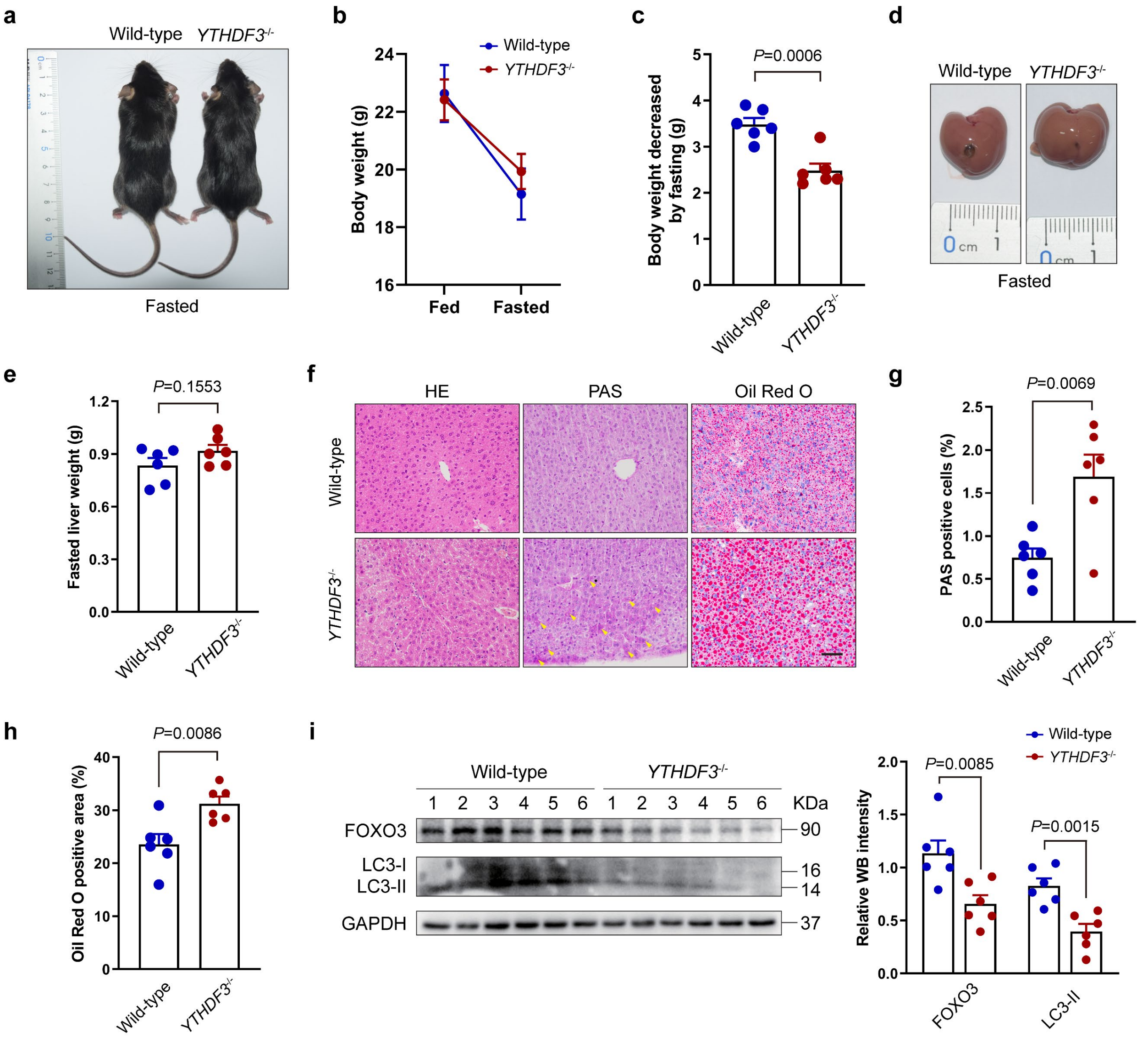

Supplementary Fig.14. The *YTHDF3*$^{-/-}$ mice show less sensitivity to starvation in vivo. a Representative image of 24 h-fasted wild-type and *YTHDF3*$^{-/-}$ mice. **b, c** Body weights were measured in wild-type and *YTHDF3*$^{-/-}$ mice before and after a 24 h fast (**b**), and the body weight changes were compared between the groups (**c**). **d** Representative image of livers from the fasted wild-type and *YTHDF3*$^{-/-}$ mice. **e** Liver weights in fasted wild-type and *YTHDF3*$^{-/-}$ mice were measured. **f** Representative images of H&E, PAS, and Oil Red O staining of liver sections from fasted wild-type and *YTHDF3*$^{-/-}$ mice. Scale bar, 50μm. **g, h** The percentages of the PAS staining positive cells (**g**) and Oil Red O staining positive areas (**h**) were compared between groups. **i** Left, immunoblot analysis of FOXO3 and LC3-II in liver tissues derived from wild-type and *YTHDF3*$^{-/-}$ mice. GAPDH is used as a loading control. Right, FOXO3 and LC3-II expressions were quantitatively defined, respectively. Data are presented as mean values ± SEM. Two-tailed unpaired *t*-tests (n=6 mice per group, male, 8 weeks old) were used to estimate significance. *P*-values are indicated in the figure. Source data are provided as a Source Data file.

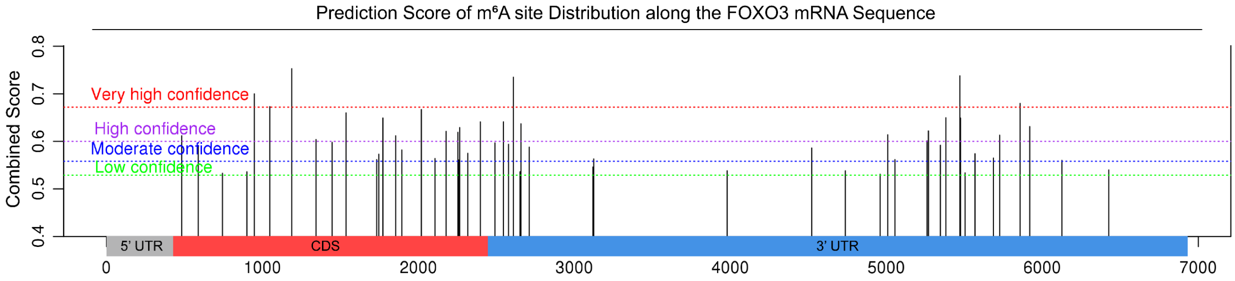

Supplementary Fig.15. Predicted m^{6}A sites in FOXO3 mRNA by the SRAMP algorithm.

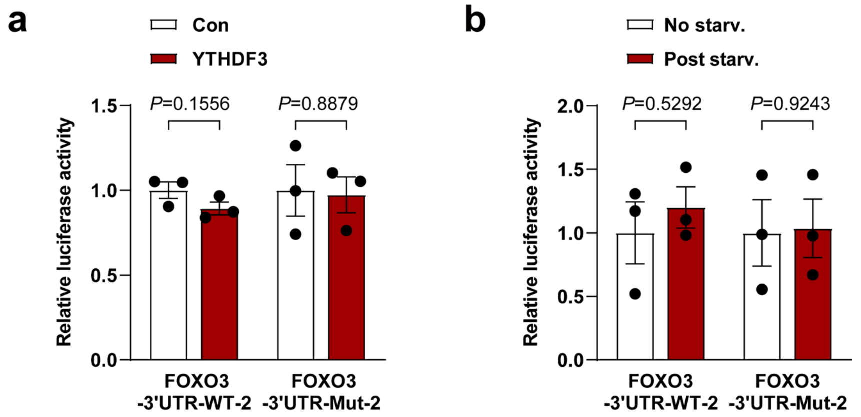

Supplementary Fig.16. Detection of the impact of the m^{6}A motifs in downstream 3' UTR on FOXO3 expression. a FOXO3-3′ UTR-WT-2 or FOXO3-3′ UTR-Mut-2 reporters were transfected into control and YTHDF3-OE MEFs for 72 hours. Firefly luciferase activity was measured and normalized to Renilla luciferase activity. **b** After transfecting FOXO3-3′UTR-WT-2 or FOXO3-3′ UTR-Mut-2, MEFs were nutrient-starved. Firefly luciferase activity was measured and normalized to Renilla luciferase activity. Data from three independent experiments are expressed as means ± SEM. Two-tailed unpaired *t*-tests were used to estimate significance. *P*-values are indicated in the figure. Source data are provided as a Source Data file.

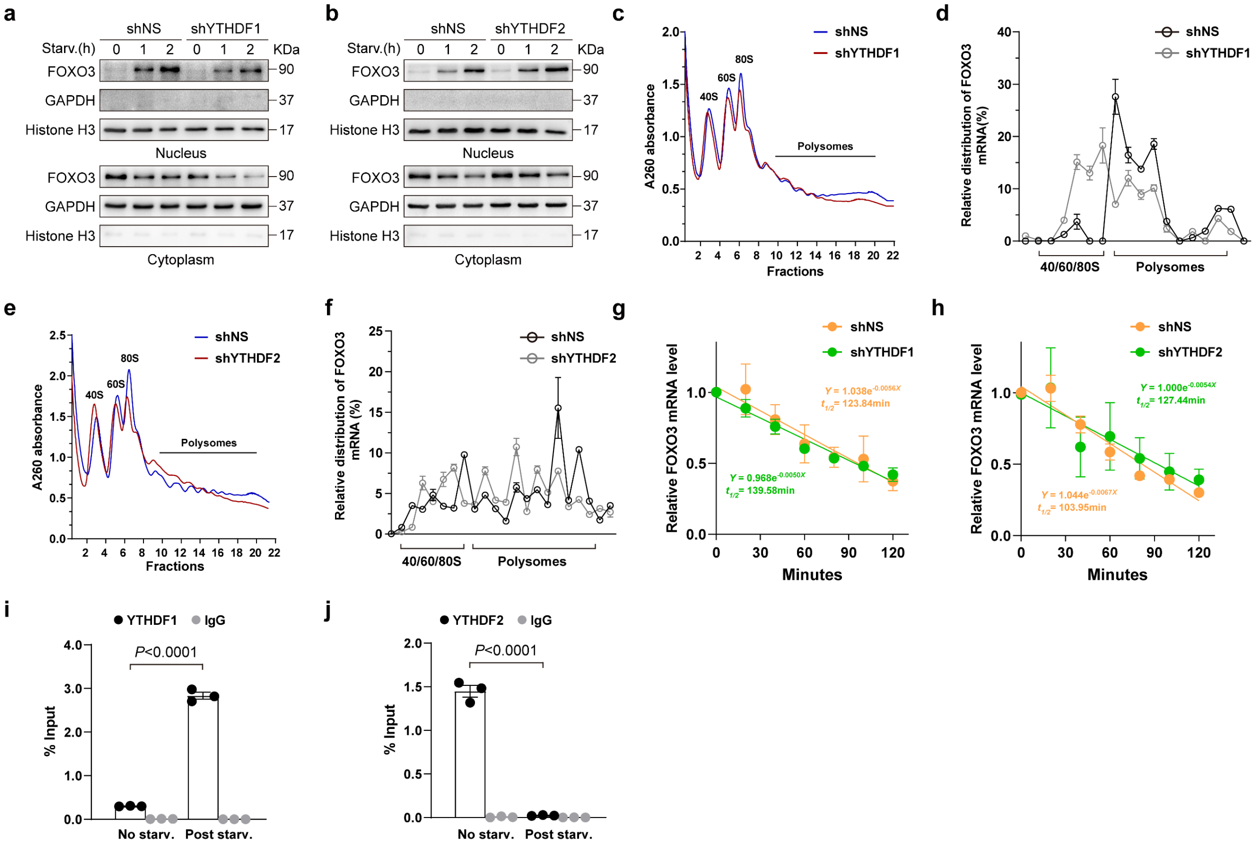

Supplementary Fig.17. Detection of the impacts of YTHDF1 or YTHDF2 depletion on FOXO3 mRNAs. a Nuclear and cytoplasm fractions from shNS and shYTHDF1 MEFs following nutrient starvation for the indicated time periods were subjected to immunoblotting. **b** Nuclear and cytoplasm fractions from shNS and shYTHDF2 MEFs following nutrient starvation for the indicated time periods were subjected to immunoblotting. **c** Sucrose gradient-based polysome profiling of shNS and shYTHDF1 MEFs. **d** FOXO3 mRNAs in each ribosome fraction were quantified through qRT-PCR and plotted as percentages of the total input from shNS and shYTHDF1 MEFs. **e** Sucrose gradient-based polysome profiling of shNS and shYTHDF2 MEFs. **f** FOXO3 mRNAs in each ribosome fraction were quantified through qRT-PCR and plotted as percentages of the total input from shNS and shYTHDF2 MEFs. **g** shNS and shYTHDF1 MEFs were treated with Act. D (5μg/mL) for the indicated time periods. The expression of FOXO3 was examined with qRT-PCR. **h** shNS and shYTHDF2 MEFs were treated with Act. D (5μg/mL) for the indicated time periods. The expression of FOXO3 was examined with qRT-PCR. **i** YTHDF1-RIP followed by qRT-PCR detected the interaction between YTHDF1 and FOXO3 mRNA in MEFs before and after nutrient starvation. **j** YTHDF2-RIP followed by qRT-PCR detected the interaction between YTHDF2 and FOXO3 mRNA in MEFs before and after nutrient starvation. Data from three independent experiments are expressed as means ± SEM. Two-tailed unpaired *t*-tests were used to estimate significance. *P*-values are indicated in the figure. Source data are provided as a Source Data file.

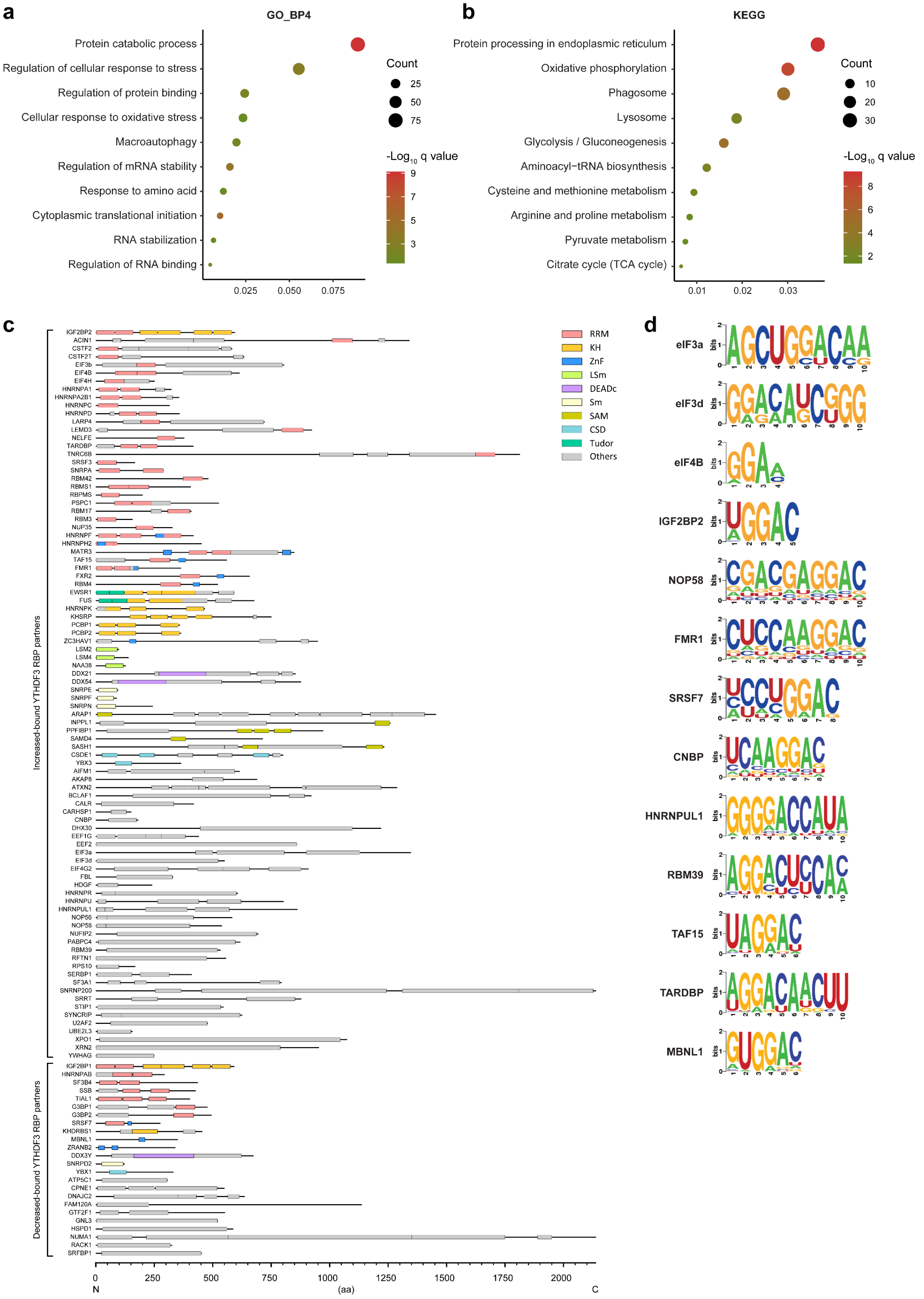
a
GO_BP4
Protein catabolic process
Regulation of cellular response to stress
Regulation of protein binding
Cellular response to oxidative stress
Macroautophagy
Regulation of mRNA stability
Response to amino acid
Cytoplasmic translational initiation
RNA stabilization
Regulation of RNA binding
Count
25
50
75
-Log10 q value
0.025
0.050
0.075
b
KEGG
Protein processing in endoplasmic reticulum
Oxidative phosphorylation
Phagosome
Lysosome
Glycolysis / Gluconeogenesis
Aminoacyl−tRNA biosynthesis
Cysteine and methionine metabolism
Arginine and proline metabolism
Pyruvate metabolism
Citrate cycle (TCA cycle)
0.01
0.02
0.03
c
Increased-bound YTHDF3 RBP partners
Decreased-bound YTHDF3 RBP partners
RRM
KH
ZnF
LSm
DEADc
Sm
SAM
CSD
Tudor
Others
N
(aa)
C
d
eIF3a
eIF3d
eIF4B
IGF2BP2
NOP58
FMR1
SRSF7
CNBP
HNRNPUL1
RBM39
TAF15
TARDBP
MBNL1

Supplementary Fig.18. YTHDF3 protein interactome data analyses. a GO biological process enrichment analysis of the 1065 proteins that were differentially bound by YTHDF3 upon nutrient deprivation. **b** KEGG pathway enrichment analysis of the 1065 proteins that were differentially bound by YTHDF3 upon nutrient deprivation. **c** Schematic representation of the domain architecture of putative YTHDF3 RBP partner proteins. The domains are named and located according to the CCD protein domain database. Specific domains are shown with boxes of the indicated colors. **d** The consensus sequence motifs identified within the indicated YTHDF3 partner's target sequences, according to the starBase and CLIPdb databases. The q-values in **a** and **b** were calculated using the ConsensusPathDB server based on hypergeometric test followed by false discovery rate (FDR) correction for multiple testing.

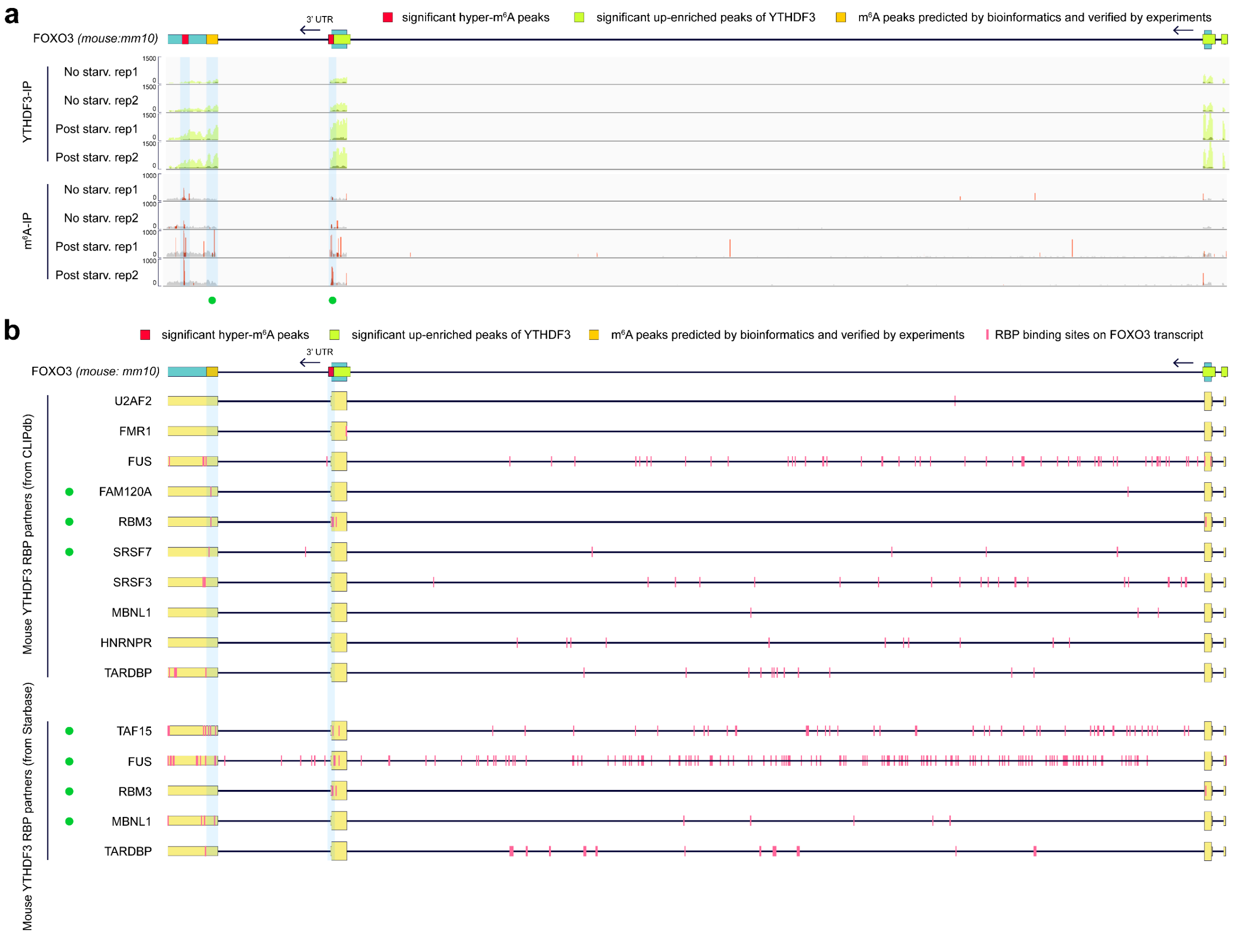

Supplementary Fig.19. IGV tracks displaying the reads of YTHDF3 RIP-seq and MeRIP-seq along the FOXO3 mRNAs. a IGV tracks displaying the reads of YTHDF3 RIP-seq (upper panels) and MeRIP-seq (lower panels) along the FOXO3 mRNAs. The red squares mark significant hyper-m^{6}A peaks from MeRIP-seq of two replicates, the green square marks significant YTHDF3 up-enriched peaks from RIP-seq of two replicates, the orange square marks the m^{6}A peaks predicted by bioinformatics, identified hyper-m^{6}A-methylated in MeRIP-seq of one replicate, and functionally verified by EMSA and dual-luciferase reporter assay. **b** IGV tracks displaying the indicated RBPs' binding sites on FOXO3 mRNAs (pink line) from CLIPdb and starBase databases mouse data. The light-blue range marks the corresponding regions to the nutrient starvation-induced hyper-m^{6}A-methylated and YTHDF3 up-enriched peaks on mouse FOXO3 transcript.

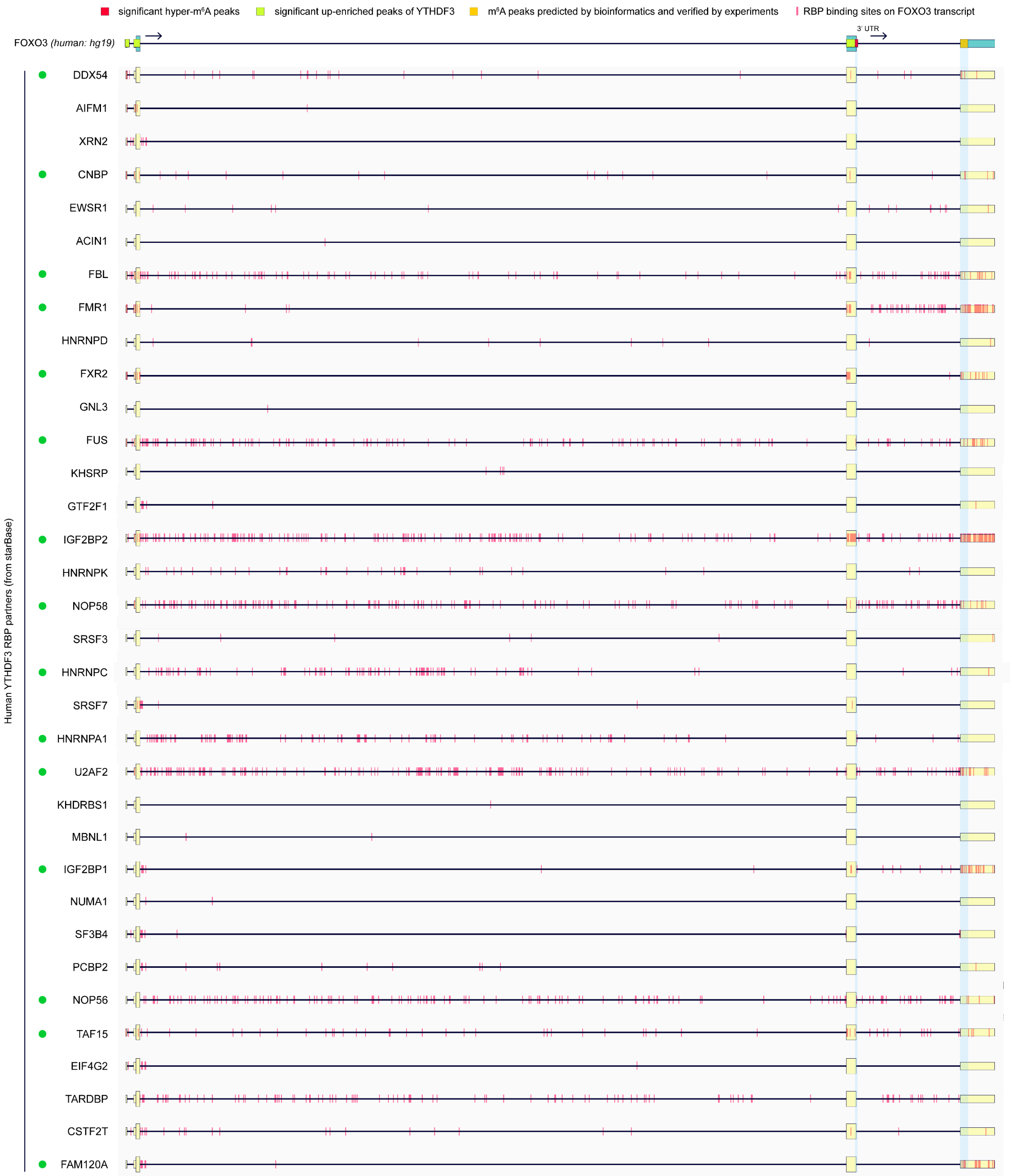

Supplementary Fig.20. IGV tracks displaying the indicated RBPs' binding sites on FOXO3 mRNAs (pink line) from the starBase human data. The light-blue ranges mark the corresponding regions to the mouse hyper-m^{6}A-methylated and YTHDF3 up-enriched peaks in response to nutrient starvation on mouse FOXO3 transcript.

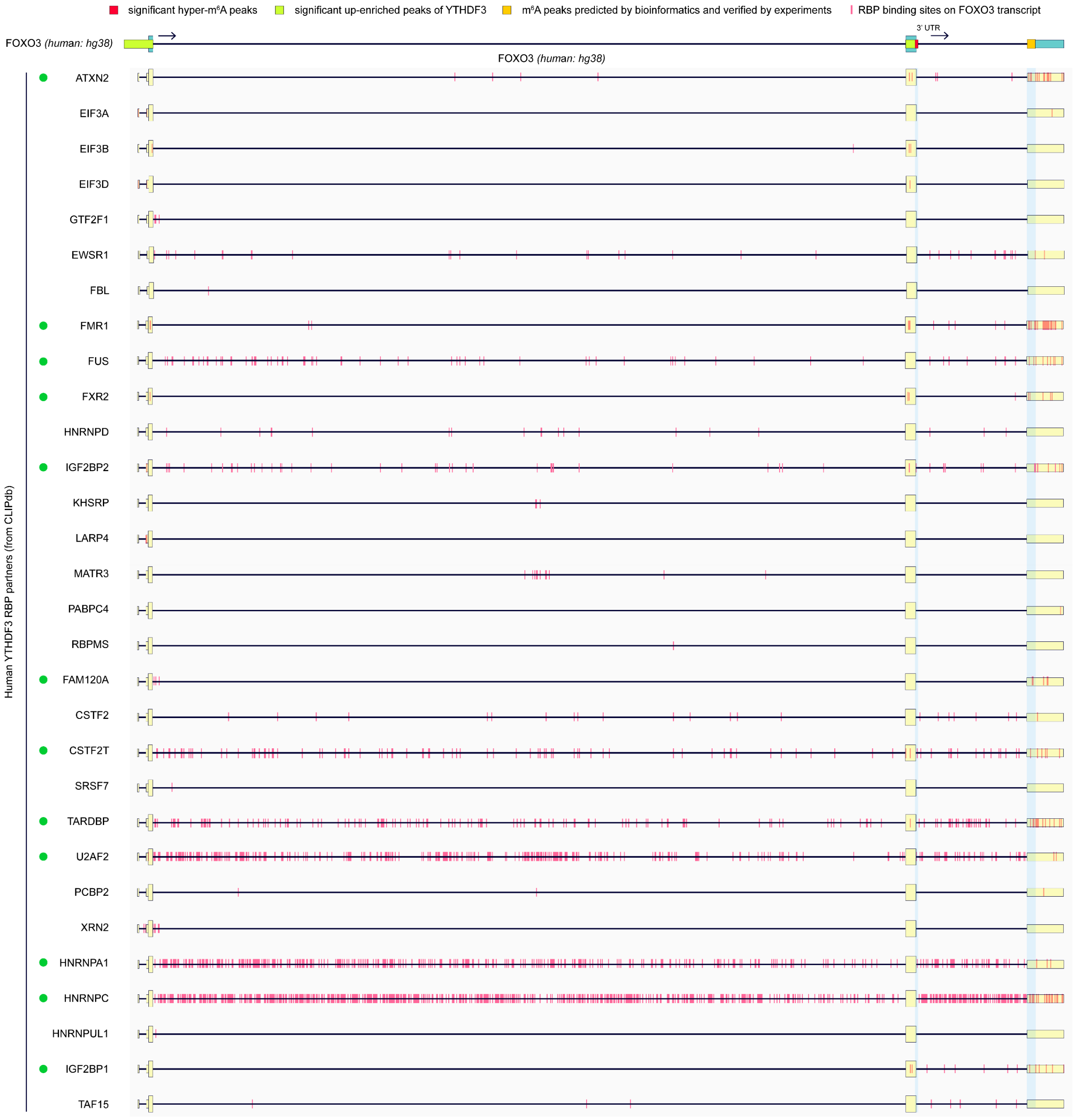

Supplementary Fig.21. IGV tracks displaying the indicated RBPs' binding sites on FOXO3 mRNAs (pink line) from CLIPdb database human data. The light-blue ranges mark the corresponding regions to the mouse hyper-m^{6}A-methylated and YTHDF3 up-enriched peaks in response to nutrient starvation on mouse FOXO3 transcript.

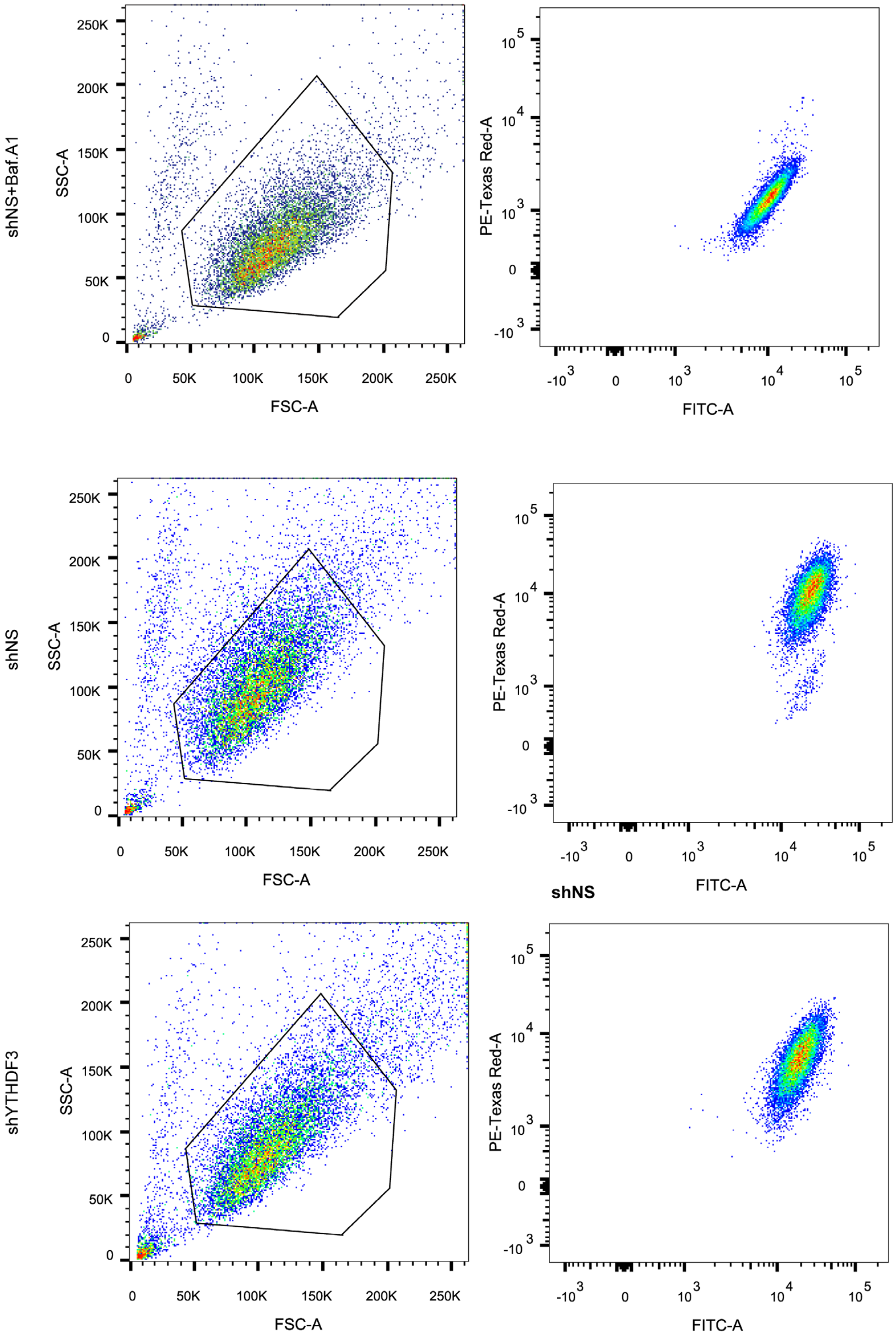

Supplementary Fig.22. Flow cytometry gating strategy. Gating strategy for AO staining in shYTHDF3 MEFs and shNS MEFs, with or without 20nM Baf.A1 for 4 hours. The left panels show cells gated on forward scatter (FSC) and side scatter (SSC) to exclude the most debris and dead cells. The right panels show cells analyzed using FITC and PE-Texas Red channels.

Supplementary Table 1. Antibodies used for this study

Antibodies	Source	Catalog No.	Dilution
METTL3	Proteintech	15073-1-AP	WB 1:1000
	Abcam	ab195352	IP 8µg per 1mg total protein
			IF 1:1000
METTL14	Proteintech	26158-1-AP	WB 1:1000
			IF 1:500
ALKBH5	Proteintech	16837-1-AP	WB 1:5000
			IF 1:500
FTO	Abcam	ab124892	WB 1:5000
	Proteintech	27226-1-AP	IF 1:300
YTHDF1	Proteintech	17479-1-AP	WB 1:1000
	Abcam	ab220161	RIP 20µg per 1mg total protein
YTHDF2	Proteintech	24744-1-AP	WB 1:1000
	Abcam	ab220163	RIP 20µg per 1mg total protein
YTHDF3	Santa Cruz Biotechnology	sc-377119	WB 1:500
			IF 1:250
			RIP 20µg per 1mg total protein
			IP 10µg per 1mg total protein
YTHDC1	ABclonal	A7318	WB 1:1000
YTHDC2	Proteintech	27779-1-AP	WB 1:1000
LC3B	Cell Signaling Technology	3868	WB 1:800
p62	Abcam	ab56416	WB 1:15000
mTOR	Cell Signaling Technology	2983	WB 1:1000
Phospho-mTOR (Ser2448)	Cell Signaling Technology	5536	WB 1:1000
4E-BP1	Cell Signaling Technology	9452	WB 1:1000
Phospho-4E-BP1 (Thr37/46)	Cell Signaling Technology	2855	WB 1:1000
p70 S6 Kinase	Cell Signaling Technology	9202	WB 1:1000

Phospho-p70 S6 Kinase (Thr389)	Cell Signaling Technology	9234	WB 1:1000
AMPKα	Cell Signaling Technology	2532	WB 1:1000
Phospho-AMPKα (Thr172)	Cell Signaling Technology	2535	WB 1:1000
Raptor (24C12)	Cell Signaling Technology	2280	WB 1:1000
Phospho-Raptor (Ser792)	Cell Signaling Technology	2083	WB 1:1000
β-actin	Proteintech	20536-1-AP	WB 1:5000
GAPDH	Proteintech	10494-1-AP	WB 1:10000
FOXO3	Santa Cruz Biotechnology	sc-48348	WB 1:1000
	Cell Signaling Technology	2497	WB 1:1000
m^{6}A	SYSY	202003	Dot blot 1:1000
			IF 1:500
Histone H3	Proteintech	17168-1-AP	WB 1:800
ULK1	Cell Signaling Technology	8054	WB 1:800
	Santa Cruz Biotechnology	sc-390904	IF 1:300
ATG13	Cell Signaling Technology	13273	WB 1:1000
			IF 1:100
ATG14	Cell Signaling Technology	96752	WB 1:1000
Phospho-ATG14	Cell Signaling Technology	92340	IF 1:600
DFCP1	Santa Cruz Biotechnology	sc-515049	WB 1:1000
			IF 1:300
eIF3A	Cell Signaling Technology	3411	WB 1:1000
eIF4B	Cell Signaling Technology	3592	WB 1:1000
ATG4A	ABclonal	A2598	WB 1:1000
ATG4C	Cell Signaling Technology	5262	WB 1:1000
ATG5	Cell Signaling Technology	12994	WB 1:1500
ATG7	Cell Signaling Technology	8558	WB 1:1000

ATG10	ABclonal	A6848	WB 1:1000
ATG12	Cell Signaling Technology	4180	WB 1:1000
ATG14	Cell Signaling Technology	96752	WB 1:1000
BECN1	Cell Signaling Technology	3495	WB 1:1000
PI3KIII	Cell Signaling Technology	4263	WB 1:1000
BNIP3	Abcam	ab109362	WB 1:1000
RAB7	Santa Cruz Biotechnology	Sc-376362	WB 1:1000
eIF4G2	Proteintech	17728-1-AP	WB 1:1000
PABP1	Proteintech	10970-1-AP	WB 1:1000
BMF	ABclonal	A5796	WB 1:1000
DDIT3	ABclonal	A0221	WB 1:1000
SESN2	Santa Cruz Biotechnology	sc-393195	WB 1:1000
ZFYVE1	Santa Cruz Biotechnology	sc-515049	WB 1:1000
CDKN1B	ABclonal	A0290	WB 1:1000
PLD6	Abcepta	AP11286c	WB 1:1000
CCR4	Affinity	DF10206	WB 1:1000
TXNIP	ABclonal	A9342	WB 1:1000
Phospho-FOXO3A(Ser413)	Affinity	AF2343	WB 1:1000
FLAG	Sigma-Aldrich	F1804	WB 1:1000<br>IP 10μg per 1mg total protein
RPS27A	Affinity	DF6761	WB 1:1000
RPL23	Proteintech	16086-1-AP	WB 1:1000
RPL11	Proteintech	16277-1-AP	WB 1:1000
Dylight 549, Goat Anti-Rabbit IgG	Abbkine	A23320	IF 1:1500
Dylight 549, Goat Anti- Mouse IgG	Abbkine	A23310	IF 1:1500
DyLight 488, Goat Anti-Mouse IgG	Abbkine	A23210	IF 1:1500
Dylight 488, Goat Anti-Rabbit IgG	Abbkine	A23220	IF 1:1500

Goat anti-Rabbit IgG (H+L)-HRP	Bioworld	BS13278	WB 1:5000
Goat anti-Mouse IgG (H+L)-HRP	Bioworld	BS12478	WB 1:5000

Supplementary Table 2. Detailed information of wild-type and two mutant FOXO3-CDS fragments

(1) FOXO3-CDS-WT:

ATGGCAGAGGCACCAGCCTCCCCGGTCCCGCTCTCTCCGCTCGAAGTGGAGCT**GGACC**CAGAGTTCGAGCCACA
GAGTCGGCCACGCTCCTGTACGTGGCCCCTGCAGAGGCCGGAGCTGCAGGCGAGCCCGGCCAAGCCCTCGGGGG
AGACGGCCGC**AGACT**CCATGATCCCCGAGGAGGACGACGATGAAGACGACGAGGACGGCGGCGGCCGAGCCAGC
TCGGCCATGGTGATCGGTGGCGGCGTGAGCAGCACGCTGGGTTCCGGGCTGCTCCTCGAGGATTCGGCCATGCT
GCTGGCTCCAGGAGGGCAGGACCTCGGGTCGGGGCCAGCGTCCGCCGCAGGCGCTCTGAGTGGGGGCACGCCGA
CGCAGCTGCAGCCTCAGCAGCCACTGCCACAGCCGCAGCCGGGGGCGGCTGGGGGCTCTGGGCAACCAAGGAAA
TGCTCCTCGCGGCGGAATGCCTGGGGGAACCTGTCCTATGCCGACCTGATCACCCGCGCCATCGAGAGCTCCCC
GGACAAACGGCTCACTTTGTCCCAGATCTACGAGTGGATGGTGCGCTGTGTGCCCTACTTCAAGGATAAGGGCG
ACAGCAACAGCTCTGCGGGCTGGAA**GAACT**CCATCCGGCACAACCTGTCCCTGCACAGCCGCTTCATGCGCGTT
CAGAATGAAGGCACGGGCAAGAGCTCTTGGTGGATCATCAACCCCGATGGGGGAAAGAGCGGGAAGGCCCCCCG
GCGGCGTGCGGTCTCCAT**GGACA**ACAGCAACAAGTACACCAAGAGCCGAGGCCGGGCAGCCAAGAAGAAGGCGG
CCCTGCAGGCTGCCCCAGAGTCGGCAGACGACAGTCCTTCCCAGCTCTCCAAGTGGCCTGGCAGCCCCACGTCC
CGCAGCAGCGACGAGCTGGATGCGT**GGACC**GACTTCCGCTCGCGCACCAATTCCAACGCCAGCACCGTGAGCGG
CCGCCTGTCGCCCATCCTGGCAAGCACGGAGCTGGATGACGTCCAGGATGATGAT**GGACC**CCTGTCCCCCATGC
TGTACAGCAGCTCTGCCAGCCTGTCGCCCTCCGTGAGCAAGCCGTGTACTGTGGAGCTTCCGCGGCTGAC**GGAC**
ATGGCCGGCACCATGAATCTGAATGATGGGCTGGCCGAGAACCTCATGGACGACCTGCTGGATAACATCGCGCT
CCCGCCATCGCAGCCATCGCCTCCTGGCGGGCTTATGCAGCGGGGCTCCAGCTTCCCATATACCGCCAAGAGCT
CCGGCCTGGGCTCCCCAACCGGCTCCTTCAACAGTACCGTGTTTGGACCTTCGTCTCTGAACTCCTTGCGTCAG
TCACCCATGCAGACTATCCAGGAGAACAGACCAGCCACCTTCTCTTCCGTGTCACACTACGGCAACCAGACACT
CCAAGACCTGCTTGCTTCAGACTCACTCAGCCACAGCGACGTCATGATGACCCAGTCGGACCCCTTGATGTCTC
AGGCTAGCACCGCCGTGTCCGCCCAGAATGCCCGCCGGAACGTGATGCTTCGCAACGATCCAATGATGTCCTTT
GCTGCCCAGCCTACCCAGGGGAGTTTGGTCAATCAGAACTTGCTCCACCACCAGCACCAAACCCAGGGCGCTCT
TGGTGGCAGCCGTGCCTTGTCAAATTCTGTCAGCAACATGGGCTTGAGTGACTCCAGCAGCCTTGGCTCAGCCA
AACACCAGCAGCAGTCTCCCGCCAGCCAGTCTATGCAAACCCTCTCGGACTCTCTCTCAGGCTCCTCACTGTAT
TCAGCTAGTGCAAACCTTCCCGTCATGGGCCACGATAAGTTCCCCAGTGACTTGGACCTGGACATGTTCAATGG
GAGCTTGGAATGTGACATGGAGTCCATCATCCGTAGTGAACTCATGGATGCTGACGGGTTGGATTTTAACTTTG
ACTCCCTCATCTCCACACAGAACGTTGTTGGTTTGAATGTGGGGAACTTCACTGGTGCTAAGCAGGCCTCATCT
CAAAGCTGGGTACCAGGCGACTACAAAGACCATGACGGTGATTATAAAGATCATGACATCGATTACAAGGATGA
CGATGACAAG

(2) FOXO3-CDS-Mut1:

ATGGCAGAGGCACCAGCCTCCCCGGTCCCGCTCTCTCCGCTCGAAGTGGAGCT**GGTCC**CAGAGTTCGAGCCACA
GAGTCGGCCACGCTCCTGTACGTGGCCCCTGCAGAGGCCGGAGCTGCAGGCGAGCCCGGCCAAGCCCTCGGGGG
AGACGGCCGC**AGTCT**CCATGATCCCCGAGGAGGACGACGATGAAGACGACGAGGACGGCGGCGGCCGAGCCAGC
TCGGCCATGGTGATCGGTGGCGGCGTGAGCAGCACGCTGGGTTCCGGGCTGCTCCTCGAGGATTCGGCCATGCT
GCTGGCTCCAGGAGGGCAGGACCTCGGGTCGGGGCCAGCGTCCGCCGCAGGCGCTCTGAGTGGGGGCACGCCGA
CGCAGCTGCAGCCTCAGCAGCCACTGCCACAGCCGCAGCCGGGGGCGGCTGGGGGCTCTGGGCAACCAAGGAAA
TGCTCCTCGCGGCGGAATGCCTGGGGGAACCTGTCCTATGCCGACCTGATCACCCGCGCCATCGAGAGCTCCCC
GGTCAAACGGCTCACTTTGTCCCAGATCTACGAGTGGATGGTGCGCTGTGTGCCCTACTTCAAGGATAAGGGCG
ACAGCAACAGCTCTGCGGGCTGGAA**GATCT**CCATCCGGCACAACCTGTCCCTGCACAGCCGCTTCATGCGCGTT
CAGAATGAAGGCACGGGCAAGAGCTCTTGGTGGATCATCAACCCCGATGGGGGAAAGAGCGGGAAGGCCCCCCG
GCGGCGTGCGGTCTCCAT**GGTCA**ACAGCAACAAGTACACCAAGAGCCGAGGCCGGGCAGCCAAGAAGAAGGCGG
CCCTGCAGGCTGCCCCAGAGTCGGCAGACGACAGTCCTTCCCAGCTCTCCAAGTGGCCTGGCAGCCCCACGTCC
CGCAGCAGCGACGAGCTGGATGCGT**GGTCC**GACTTCCGCTCGCGCACCAATTCCAACGCCAGCACCGTGAGCGG
CCGCCTGTCGCCCATCCTGGCAAGCACGGAGCTGGATGACGTCCAGGATGATGAT**GGTCC**CCTGTCCCCCATGC
TGTACAGCAGCTCTGCCAGCCTGTCGCCCTCCGTGAGCAAGCCGTGTACTGTGGAGCTTCCGCGGCTGAC**GGTC**
ATGGCCGGCACCATGAATCTGAATGATGGGCTGGCCGAGAACCTCATGGACGACCTGCTGGATAACATCGCGCT
CCCGCCATCGCAGCCATCGCCTCCTGGCGGGCTTATGCAGCGGGGCTCCAGCTTCCCATATACCGCCAAGAGCT
CCGGCCTGGGCTCCCCAACCGGCTCCTTCAACAGTACCGTGTTTGGACCTTCGTCTCTGAACTCCTTGCGTCAG
TCACCCATGCAGACTATCCAGGAGAACAGACCAGCCACCTTCTCTTCCGTGTCACACTACGGCAACCAGACACT
CCAAGACCTGCTTGCTTCAGACTCACTCAGCCACAGCGACGTCATGATGACCCAGTCGGACCCCTTGATGTCTC
AGGCTAGCACCGCCGTGTCCGCCCAGAATGCCCGCCGGAACGTGATGCTTCGCAACGATCCAATGATGTCCTTT
GCTGCCCAGCCTACCCAGGGGAGTTTGGTCAATCAGAACTTGCTCCACCACCAGCACCAAACCCAGGGCGCTCT
TGGTGGCAGCCGTGCCTTGTCAAATTCTGTCAGCAACATGGGCTTGAGTGACTCCAGCAGCCTTGGCTCAGCCA

AACACCAGCAGCAGTCTCCCGCCAGCCAGTCTATGCAAACCCTCTCGGACTCTCTCTCAGGCTCCTCACTGTAT
TCAGCTAGTGCAAACCTTCCCGTCATGGGCCACGATAAGTTCCCCAGTGACTTGGACCTGGACATGTTCAATGG
GAGCTTGGAATGTGACATGGAGTCCATCATCCGTAGTGAACTCATGGATGCTGACGGGTTGGATTTTAACTTTG
ACTCCCTCATCTCCACACAGAACGTTGTTGGTTTGAATGTGGGGAACTTCACTGGTGCTAAGCAGGCCTCATCT
CAAAGCTGGGTACCAGGCGACTACAAAGACCATGACGGTGATTATAAAGATCATGACATCGATTACAAGGATGA
CGATGACAAGTGA

(3) FOXO3-CDS-Mut2:

ATGGCAGAGGCACCAGCCTCCCCGGTCCCGCTCTCTCCGCTCGAAGTGGAGCTGGACCCAGAGTTCGAGCCACA
GAGTCGGCCACGCTCCTGTACGTGGCCCCTGCAGAGGCCGGAGCTGCAGGCGAGCCCGGCCAAGCCCTCGGGGG
AGACGGCCGCAGACTCCATGATCCCCGAGGAGGACGACGATGAAGACGACGAGGACGGCGGCGGCCGAGCCAGC
TCGGCCATGGTGATCGGTGGCGGCGTGAGCAGCACGCTGGGTTCCGGGCTGCTCCTCGAGGATTCGGCCATGCT
GCTGGCTCCAGGAGGGCAGGACCTCGGGTCGGGGCCAGCGTCCGCCGCAGGCGCTCTGAGTGGGGGCACGCCGA
CGCAGCTGCAGCCTCAGCAGCCACTGCCACAGCCGCAGCCGGGGGCGGCTGGGGGCTCTGGGCAACCAAGGAAA
TGCTCCTCGCGGCGGAATGCCTGGGGGAACCTGTCCTATGCCGACCTGATCACCCGCGCCATCGAGAGCTCCCC
GGACAAACGGCTCACTTTGTCCCAGATCTACGAGTGGATGGTGCGCTGTGTGCCCTACTTCAAGGATAAGGGCG
ACAGCAACAGCTCTGCGGGCTGGAAGAACTCCATCCGGCACAACCTGTCCCTGCACAGCCGCTTCATGCGCGTT
CAGAATGAAGGCACGGGCAAGAGCTCTTGGTGGATCATCAACCCCGATGGGGGAAAGAGCGGGAAGGCCCCCCG
GCGGCGTGCGGTCTCCATGGACAACAGCAACAAGTACACCAAGAGCCGAGGCCGGGCAGCCAAGAAGAAGGCGG
CCCTGCAGGCTGCCCCAGAGTCGGCAGACGACAGTCCTTCCCAGCTCTCCAAGTGGCCTGGCAGCCCCACGTCC
CGCAGCAGCGACGAGCTGGATGCGTGGACCGACTTCCGCTCGCGCACCAATTCCAACGCCAGCACCGTGAGCGG
CCGCCTGTCGCCCATCCTGGCAAGCACGGAGCTGGATGACGTCCAGGATGATGATGGACCCCTGTCCCCCATGC
TGTACAGCAGCTCTGCCAGCCTGTCGCCCTCCGTGAGCAAGCCGTGTACTGTGGAGCTTCCGCGGCTGACGGAC
ATGGCCGGCACCATGAATCTGAATGATGGGCTGGCCGAGAACCTCATGGACGACCTGCTGGATAACATCGCGCT
CCCGCCATCGCAGCCATCGCCTCCTGGCGGGCTTATGCAGCGGGGCTCCAGCTTCCCATATACCGCCAAGAGCT
CCGGCCTGGGCTCCCCAACCGGCTCCTTCAACAGTACCGTGTTTGGTCCTTCGTCTCTGATCTCCTTGCGTCAG
TCACCCATGCAGTCTATCCAGGAGAACAGACCAGCCACCTTCTCTTCCGTGTCACACTACGGCAACCAGACACT
CCAAGACCTGCTTGCTTCAGTCTCACTCAGCCACAGCGACGTCATGATGACCCAGTCGGTCCCCTTGATGTCTC
AGGCTAGCACCGCCGTGTCCGCCCAGAATGCCCGCCGGAACGTGATGCTTCGCAACGATCCAATGATGTCCTTT
GCTGCCCAGCCTACCCAGGGGAGTTTGGTCAATCA**GATCT**TGCTCCACCACCAGCACCAAACCCAGGGCGCTCT
TGGTGGCAGCCGTGCCTTGTCAAATTCTGTCAGCAACATGGGCTTGAG**TGTCT**CCAGCAGCCTTGGCTCAGCCA
AACACCAGCAGCAGTCTCCCGCCAGCCAGTCTATGCAAACCCTCTC**GGTCT**CTCTCTCAGGCTCCTCACTGTAT
TCAGCTAGTGCAAACCTTCCCGTCATGGGCCACGATAAGTTCCCCAG**TGTCT**T**GGTCC**T**GGTCA**TGTTCAATGG
GAGCTTGGAATGTGACATGGAGTCCATCATCCGTAGT**GATCT**CATGGATGCTGACGGGTTGGATTTTAACTTTG
ACTCCCTCATCTCCACACAGAACGTTGTTGGTTTGAATGTGGG**GATCT**TCACTGGTGCTAAGCAGGCCTCATCT
CAAAGCTGGGTACCAGGCGACTACAAAGACCATGACGGTGATTATAAAGATCATGACATCGATTACAAGGATGA
CGATGACAAG

Supplementary Table 3. List of primer pairs used for qRT-PCR analysis

Gene	Forward Primer (5'-3')	Reverse Primer (5'-3')
GAPDH	CATGGCCTTCCGTGTTCCTA	CCTGCTTCACCACCTTCTTG
BECN1	ATGGAGGGGTCTAAGGCGTC	TCCTCTCCTGAGTTAGCCTCT
YTHDF3	GATCAGCCTATGCCATATCTGAC	CCCCTGGTTGACTAAAAACACC
BNIP3	TCCTGGGTAGAACTGCACTTC	GCTGGGCATCCAACAGTATTT
ATG4A	GCTGGTATGGATTCTGGGGAA	TGGGTTGTTCTTTTTGTCTCTCC
ATG4C	AGATGAAAGCAAGATGTTGCCT	CCCTGTAGGTCAGCCATATTCTA
ATG5	AGCCAGGTGATGATTCACGG	GGCTGGGGGACAATGCTAA
ATG7	GTTCGCCCCCTTTAATAGTGC	TGAACTCCAACGTCAAGCGG
ATG10	GTAGTTACCAAGTGCCGGTTC	AGCTAACGGTCTCCCATCTAAA
ATG12	CCAAGGACTCATTGACTTCATC	CAGTAATGCAGGACCAGTTTACC
ATG13	CCAGGCTCGACTTGGAGAAAA	AGATTTCCACACACATAGATCGC
ATG14	GAGGGCCTTTACGTGGCTG	AATAGACGAAATCACCGCTCTG
ULK1	AAGTTCGAGTTCTCTCGCAAG	CGATGTTTTCGTGCTTTAGTTCC
RAB7	GGTTCCGCTCAATGGTATCAA	TGCCCTGGTACTTCCTATCTTC
PI3KIII	AAAGGCCGAGCCCTCTATTAT	GGACAATCTCGACGTAAGAAGC
ZFYVE26	AACTGGGCGTTGAGGTCTCTA	CAGCGAGTGAACTTCTCCCTT
BMF	GGAGCGGGCGTATTTTGGAA	ACACTCGATTGGGAAGAAGGG
DDIT3	CTGGAAGCCTGGTATGAGGAT	CTGGAAGCCTGGTATGAGGAT
AP4M1	CCCGGTTGTCATGTATCACGG	AGCCACAGTAGTCACCCAAGA
SESN2	TCCGAGTGCCATTCCGAGAT	TCCGGGTGTAGACCCATCAC
ZFYEV1	CATGTGCCAGGAGAGTTATGC	GAGTCTTATCCGCTCATGGTTTC
Firefly luciferase	ATCCGGAAGCGACCAACGCC	GTCGGGAAGACCTGCCACGC
FOXO3	CTGGGGGAACCTGTCCTATG	TCATTCTGAACGCGCATGAAG
FOXO3-3'UTR-m^6A	GCCTTCGCCATCCTAGCTCT	GTTCTATGTGCTTCAGCCGTCA

FOXO3-CDS-m^{6}A	ATGGATGCTGACGGGTTGGA	GTACCCAGCTTTGAGATGAGG
FOXO3-NC	TGCCTTTCCCAAGCTGCTAC	CAGGTCACACGACTCCTATACAC

Supplementary Table 4. Biotin-m^{6}A oligonucleotides used in this study

**Name**	**Sequences (5’-3’)**
Foxo3-CDS-biotin-RNAProbe 1	biotin-GUGACUUGGACCUGGAC
Foxo3-CDS-biotin-RNAProbe 2	biotin-CCGUAGUGAACUCAUG
Foxo3-CDS-biotin-RNAProbe 3	biotin-GGGGAACUUCACUGG
Foxo3-CDS-m^{6}A-biotin-RNAProbe 1	biotin-GUG**A*(m^{6}A)**CUUGG**A*(m^{6}A)**CCUGG**A*(m^{6}A)**C
Foxo3-CDS-m^{6}A-biotin-RNAProbe 2	biotin-CCGUAGUGA**A*(m^{6}A)**CUCAUG
Foxo3-CDS-m^{6}A-biotin-RNAProbe 3	biotin-GGGGA**A*(m^{6}A)**CUUCACUGG
Foxo3-3’UTR-biotin-RNAProbe 4	biotin-CAAGUGGACAGUGAUCC
Foxo3-3’UTR-biotin-RNAProbe 5	biotin-CCCAGCAGAGACUGUUA
Foxo3-3’UTR-biotin-RNAProbe 6	biotin-GGAACAGAACUCUAUAA
Foxo3-3’UTR-m^{6}A-biotin-RNAProbe 4	biotin-CAAGUGG**A*(m^{6}A)**CAGUGAUCC
Foxo3-3’UTR-m^{6}A-biotin-RNAProbe 5	biotin-CCCAGCAGAG**A*(m^{6}A)**CUGUUA
Foxo3-3’UTR-m^{6}A-biotin-RNAProbe 6	biotin-GGAACAGA**A*(m^{6}A)**CUCUAUAA

Supplementary Table 5. Sequences of siRNA used in the study

**Target gene**	**siRNA sequence (5'-3')**
siFOXO3	CCGTGGAACAGAACTCTAT
siRPS27a	GACTTACTGCTTCAACAAA
siRPS27a-2	GCCACTTTGACAGGCATTA
siRPL23	GAAAGGCAAACCAGAACTA
siRPL23-2	CAACGAAAGTCATATCGAA
siRPL11	CCGGAGAAATGAGAAGATT
