# Supplementary material for: Autophagy induction promoted by m6A reader YTHDF3 through translation upregulation of FOXO3 mRNA
Source: Nat Commun. 2022 Oct 4;13:5845. doi: 10.1038/s41467-022-32963-0 (PMC9532426; doi:10.1038/s41467-022-32963-0)
Supplement: Supplementary file 3 — Reporting Summary [file 41467_2022_32963_MOESM3_ESM.pdf]

Corresponding author(s): Jia-Hong Wang,Yan Sun,Dong Xiao

Last updated by author(s): 2022-7-23

Reporting Summary

Nature Portfolio wishes to improve the reproducibility of the work that we publish. This form provides structure for consistency and transparency in reporting. For further information on Nature Portfolio policies, see our Editorial Policies and the Editorial Policy Checklist.

Statistics

For all statistical analyses, confirm that the following items are present in the figure legend, table legend, main text, or Methods section.

n/a	Confirmed	
☐	☒	The exact sample size (*n*) for each experimental group/condition, given as a discrete number and unit of measurement
☐	☒	A statement on whether measurements were taken from distinct samples or whether the same sample was measured repeatedly
☐	☒	The statistical test(s) used AND whether they are one- or two-sided<br>*Only common tests should be described solely by name; describe more complex techniques in the Methods section.*
☐	☒	A description of all covariates tested
☐	☒	A description of any assumptions or corrections, such as tests of normality and adjustment for multiple comparisons
☐	☒	A full description of the statistical parameters including central tendency (e.g. means) or other basic estimates (e.g. regression coefficient) AND variation (e.g. standard deviation) or associated estimates of uncertainty (e.g. confidence intervals)
☐	☒	For null hypothesis testing, the test statistic (e.g. *F*, *t*, *r*) with confidence intervals, effect sizes, degrees of freedom and *P* value noted<br>*Give P values as exact values whenever suitable.*
☒	☐	For Bayesian analysis, information on the choice of priors and Markov chain Monte Carlo settings
☒	☐	For hierarchical and complex designs, identification of the appropriate level for tests and full reporting of outcomes
☒	☐	Estimates of effect sizes (e.g. Cohen's *d*, Pearson's *r*), indicating how they were calculated

Our web collection on statistics for biologists contains articles on many of the points above.

Software and code

Policy information about availability of computer code

Data collection	The blots were imaged with the ChemiDoc'M XRS+ imagin g system (Bio -Rad). The confocal im ages were visualized and collected using a Nikon Al confocal microscope. The TEM images were observed and collected under a Hitachi-7500 electron microscope. The LC-MS-based proteomics analysis were performed on the Orbitrap Fusion Tribrid LC mass spectrometer (Thermo). The RIP-seq was performed with an lllumina Hiseq platform. The MeRIP-seq reads were harvested from an lllumina HiSeq 4000 sequencer.
Data analysis	MaxQuant (vl .5.8.3), Perseus (vl.5.4.1), Trimmomatic (v0 .36), Tophat (v2.0.13), diff Reps (v1.55.6), HOMER (v4.9.1), Guitar Bioconductor package (v1.20.1), Cutadapt (vl.9.3), HISAT 2 (v2.0.4), MACS2 (v2.1.1), DREME(v5.4.1), Phyre2, Pymol (v2.2.0), lmageJ (v2.0.0), FlowJo (v10.5), Graphpad Prism 9.

For manuscripts utilizing custom algorithms or software that are central to the research but not yet described in published literature, software must be made available to editors and reviewers. We strongly encourage code deposition in a community repository (e.g. GitHub). See the Nature Portfolio guidelines for submitting code & software for further information.

Data

Policy information about availability of data

All manuscripts must include a data availability statement. This statement should provide the following information, where applicable:

- Accession codes, unique identifiers, or web links for publicly available datasets
- A description of any restrictions on data availability
- For clinical datasets or third party data, please ensure that the statement adheres to our policy

All data needed to evaluate the conclusions in the paper are present in the manuscription and the Supplementary Information. The accession number for the RIP -seq and MeRIP-seq reported in this manuscription is GEO: GSE158660 (secure token: mpgtmigkxdmvtar) and GSE158268(secure token: gtwxkmooxdyzxqr). The mass spectrometry proteomics data have been deposited to the ProteomeXchange Consortium (http://proteomecentral.proteomexchange.org) via the iPro X partner repository with the dataset identifier PXD025450. Raw data that support the findings of this study are available from the corresponding author (D.X.) upon reasonable request.

Human research participants

Policy information about studies involving human research participants and Sex and Gender in Research.

Reporting on sex and gender	No human researcher participants
Population characteristics	No human researcher participants
Recruitment	No human researcher participants
Ethics oversight	No human researcher participants

Note that full information on the approval of the study protocol must also be provided in the manuscript.

Field-specific reporting

Please select the one below that is the best fit for your research. If you are not sure, read the appropriate sections before making your selection.

☒ Life sciences ☐ Behavioural & social sciences ☐ Ecological, evolutionary & environmental sciences

For a reference copy of the document with all sections, see nature.com/documents/nr-reporting-summary-flat.pdf

Life sciences study design

All studies must disclose on these points even when the disclosure is negative.

Sample size	Each experiment was repeated three times independently unless otherwise specified.
Data exclusions	No data were excluded.
Replication	Experiments in cells in culture were performed on different days to confirm reproducibility of the procedures. All independent replications were successful.
Randomization	For the studies involving cells in culture treatment groups were attributed randomly between wells and plates to account for well or tube positioning effects.
Blinding	Most of the experiments were performed by different co-authors. The data were also processed and re-evaluated by more than two authors.

Reporting for specific materials, systems and methods

We require information from authors about some types of materials, experimental systems and methods used in many studies. Here, indicate whether each material, system or method listed is relevant to your study. If you are not sure if a list item applies to your research, read the appropriate section before selecting a response.

Materials & experimental systems

n/a	Involved in the study
☐	☒ Antibodies
☐	☒ Eukaryotic cell lines
☒	☐ Palaeontology and archaeology
☐	☒ Animals and other organisms
☒	☐ Clinical data
☒	☐ Dual use research of concern

Methods

n/a	Involved in the study
☒	☐ ChIP-seq
☐	☒ Flow cytometry
☒	☐ MRI-based neuroimaging

Antibodies

Antibodies used

Primary antibodies:
anti-METTL3, Proteintech,Cat#15073-1-AP,WB dilution 1:1000;
anti-METTL3(clone EPR18810), Abcam,Cat#ab195352, IP dilution 8μg per 1mg total protein;
anti-METTL14, Proteintech,Cat#26158-1-AP,IF dilution 1:500,WB dilution 1:1000;
anti-ALKBH5,Proteintech,Cat#16837-1-AP,WB dilution 1:5000,IF dilution 1:500;
anti-FTO(clone EPR6895),Abcam,Cat#ab124892,WB dilution 1:5000;anti-FTO,Proteintech,Cat#27226-1-AP,IF dilution 1:300;
anti-YTHDF1,Proteintech,Cat#17479-1-AP,WB dilution 1:1000;anti-YTHDF1(clone EPR22349-41),Abcam,Cat#ab220162,RIP dilution 20μg per 1mg total protein;
anti-YTHDF2,Proteintech,Cat#24744-1-AP,WB dilution 1:1000;anti-YTHDF2(clone EPR20318),Abcam,Cat#ab220163,RIP dilution 20μg per 1mg total protein;
anti-YTHDF3,Santa Cruz,Cat#2sc-377119,WB dilution 1:500,IF dilution 1:250,IP dilution 10μg per 1mg total protein, RIP dilution 20μg per 1mg total protein;
anti-YTHDC1,ABclonal,Cat#A7318,WB dilution1:1000;anti-YTHDC2,Proteintech,Cat#27779-1-AP,WB dilution1:1000;
anti-LC3B(clone D11),Cell Signaling Technology,Cat#3868S,WB dilution1:800;anti-p62(clone 2C11),Abcam,Cat#ab56416,WB dilution1:15000;
anti-mTOR(clone 7C10),Cell Signaling Technology,Cat#,2983,WB dilution1:1000;anti-Phospho-mTOR (Ser2448)(clone D9C2),Cell Signaling Technology,Cat#5536,WB dilution1:1000;
anti-4E-BP1,Cell Signaling Technology,Cat#9452,WB dilution1:1000;anti-Phospho-4E-BP1(Thr37/46)(clone 236B4),Cell Signaling Technology,Cat#2855,WB dilution1:1000;
anti-p70 S6 Kinase,Cell Signaling Technology,Cat#9202,WB dilution1:1000;
anti- Phospho-p70 S6 Kinase (Thr389)(clone 108D2),Cell Signaling Technology,Cat#9234, WB dilution1:1000;anti-4E-BP1,Cell Signaling Technology,Cat#9452,WB dilution1:1000;
anti-Phospho-4E-BP1(Thr37/46)(clone 236B4),Cell Signaling Technology,Cat#2855,WB dilution1:1000;anti-p70 S6 Kinase,Cell Signaling Technology,Cat#9202,WB dilution1:1000;
anti-Phospho-p70 S6 Kinase(Thr389)(clone 108D2),Cell Signaling Technology,Cat#9234, dilution1:1000;anti-AMPKα,Cell Signaling Technology,Cat#2532, dilution1:1000;
anti-Phospho-AMPKα (Thr172)(clone 40H9),Cell Signaling Technology,Cat#2535, WB dilution1:1000; anti-Raptor (24C12),Cell Signaling Technology,Cat#2280,WB dilution1:1000;
anti-Phospho-Raptor (Ser792),Cell Signaling Technology,Cat#2083,dilution1:1000;anti-β-actin,Proteintech,Cat#20536-1-AP,dilution1:5000;
anti-GAPDH,Proteintech,Cat#10494-1-AP,WB dilution1:10000;anti-FOXO3A,Cell Signaling Technology,Cat#2497,WB dilution1:1:1000;
anti- m6A,SYSY,Cat#202003,dot blot dilution1:1000,IF dilution 1:500;anti-Histone H3(clone D1H2),Proteintech,Cat#17168-1-AP,WB dilution1:800;
anti-ULK1,Cell Signaling Technology,Cat#8054,WB dilution1:800;anti-ULK1,Santa Cruz,Cat#sc-390904,IF dilution 1:300;
anti-ATG13(clone D4P1K),Cell Signaling Technology,Cat#13273,WB dilution1:1000,IF dilution 1:100;anti-ATG14(clone D1A1N),Cell Signaling Technology,Cat#96752,WB dilution 1:1000;
anti-Phospho-ATG14(clone D4B8M),Cell Signaling Technology,Cat#92340,IF dilution1:600;anti-DFCP1,Santa Cruz,Cat#sc-515049,WB dilution1:1000;
anti-eIF3a(clone D51F4),Cell Signaling Technology,Cat#3411,WB dilution1:1000;anti-eIF4B,Cell Signaling Technology,Cat#3592,WB dilution1:1000;
anti-ATG4A,ABclonal,Cat#A2598,WB dilution1:1000;anti-ATG4C,Cell Signaling Technology,Cat#5262,WB dilution1:1000;
anti-ATG5(clone D5F5U),Cell Signaling Technology,Cat#12994,WB dilution1:1500;anti-ATG7(clone D12B11),Cell Signaling Technology,Cat#8558,WB dilution1:1000;
anti-ATG10,ABclonal,Cat#A6848,dilution1:1000;anti-ATG12(clone D88H11), Cell Signaling Technology,Cat#4180,dilution1:1000;
anti-ATG14(clone D1A1N), Cell Signaling Technology,Cat#96752,WB dilution1:1000;anti-BECN1(clone D40C5),Cell Signaling Technology,Cat#3495, WB dilution1:1000;
anti-PI3K?(clone D9A5),Cell Signaling Technology,Cat#4263, WB dilution1:1000;anti-BNIP3(clone EPR4034), Abcam, Cat#ab109362, WB dilution1:1000;
anti-RAB7,Santa Cruz, Cat#Sc-376362,dilution1:1000;anti-eIF4G2, Proteintech, Cat#17728-1-AP, dilution1:1000;
anti-PABP1, Proteintech, Cat#10970-1-AP, WB dilution1:1000;anti-BMF, ABclonal, Cat#A5796, WB dilution1:1000;
anti-DDIT3, ABclonal, Cat#A0221, dilution1:1000;anti-SESN2(clone EPR18907), Abcam, Cat#ab178518, dilution1:1000;
anti-ZFYVE1, Santa Cruz, Cat#sc-515049, WB dilution1:1000,IF dilution 1:300;anti-CDKN1B, ABclonal, Cat#A0290, WB dilution1:1000;
anti-PLD6, Abcepta, Cat# AP11286c, WB dilution1:1000;anti-CCR4, Affinity, Cat# DF10206, WB dilution1:1000;
anti-TXNIP, ABclonal, Cat# A9342, WB dilution1:1000;anti-TNIP2, ABclonal, Cat# A4962, WB dilution1:1000;
anti-Phospho-FOXO3A(Ser413), Affinity, Cat# AF2343, WB dilution1:1000;anti-FLAG, Sigma-Aldrich, Cat#F1804, WB dilution1:1000,IP dilution 10μg per 1mg total protein;
anti-RPS27A, Affinity, Cat#DF6761, dilution1:1000;anti-RPL23, Proteintech, Cat#16086-1-AP, WB dilution 1:1000;
anti-RPL11, Proteintech, Cat#16277-1-AP, WB dilution1:1000;
Secondary antibodies:

	Dylight 549, Goat Anti-Rabbit IgG,Abbkine, Cat#A23320, IF dilution1:1500;<br>Dylight 549, Goat Anti-Mouse IgG,Abbkine, Cat#A23310, IF dilution1:1500;<br>DyLight 488, Goat Anti-Mouse IgG,Abbkine, Cat#A23210, IF dilution1:1500;<br>Dylight 488, Goat Anti-Rabbit IgG,Abbkine, Cat#A23220, IF dilution1:1500;<br>Goat anti-Rabbit IgG (H+L)-HRP,Bioworld, Cat#BS13278, WB dilution1:5000;<br>Goat anti-Mouse IgG (H+L)-HRP,Bioworld, Cat#BS12478, WB dilution1:5000.
Validation	All commercial antibodies were validated for the species and applications for which they were used in this study, and used according to manufacturer's instructions.

Eukaryotic cell lines

Policy information about cell lines and Sex and Gender in Research

Cell line source(s)	MEFs were isolated from mouse embryos at 13.5 days post coitum and cultured accordin g to standard conditions in our laboratory.HEK293T (SCSP-502), 3T3-L1 (SCSP-5038), C2C12 (SCSP-505), and HepG2 (SCSP-510) cell lines were obtained from the Cell Bank of Shanghai Institute of Cell Biology, Chinese Academy of Sciences (Shanghai, China).
Authentication	HEK 293T and HepG2 cell lines were authenticated by STR profilling. Primary MEFs were authenticated by morphology.
Mycoplasma contamination	All the cell lines were tested for mycoplasma contamination using MycoBlue Mycoplasma Detector kit (D101-01,Vazyme) and all of them tested negative.
Commonly misidentified lines<br>(See ICLAC register)	No cell lines used are listed in the database of commonly misidentified cell lines.

Animals and other research organisms

Policy information about studies involving animals; ARRIVE guidelines recommended for reporting animal research, and Sex and Gender in Research

Laboratory animals	Wild-type C57BL/6 or YTHDF3+/- female mice at E13.5 gestation, and age-matched wild-type and YTHDF3-/- male mice at 8 weeks old were uesd in the indicated studies.Housing condition: Temperature 23±2?, humidity (high/low %) 37/21, dark/light cycle 6pm-6am.
Wild animals	No wild animal were used in the study.
Reporting on sex	The animal data generated here was analyzed not considering sex as a variable
Field-collected samples	No field-collected samples were used in the study.
Ethics oversight	Animal experiments were conducted in strict accordance with Southern Medical University guidelines for the Care and Use of Experimental Animals.The animals program was approved by the Animal Experimental Ethics Committee of Southern Medical University (L2022097). All surgeries were performed under pentobarbital sodium anesthesia, and every effort was made to minimize animals suffering.

Note that full information on the approval of the study protocol must also be provided in the manuscript.

Flow Cytometry

Plots

Confirm that:

☒ The axis labels state the marker and fluorochrome used (e.g. CD4-FITC).

☒ The axis scales are clearly visible. Include numbers along axes only for bottom left plot of group (a 'group' is an analysis of identical markers).

☒ All plots are contour plots with outliers or pseudocolor plots.

☒ A numerical value for number of cells or percentage (with statistics) is provided.

Methodology

Sample preparation	Cells were preloaded with 1μg/ml acridine orange(AO, aladdin) for 15 miutes at 37??followed by three fast washes.
Instrument	Fortessa flow cytometer (BD)
Software	FlowJo v10.5
Cell population abundance	Thousands of cells were evaluated

Gating strategy

After FSC/SSC gating, cells were analyzed under green and red channels as described in the Methods section.

☒ Tick this box to confirm that a figure exemplifying the gating strategy is provided in the Supplementary Information.
